# ﻿A new species of the genus *Rhaphidosoma* Amyot et Serville, 1843 (Heteroptera, Reduviidae), with data on its chromosome complement

**DOI:** 10.3897/CompCytogen.v15.i4.78718

**Published:** 2021-12-15

**Authors:** Dmitry A. Gapon, Valentina G. Kuznetsova, Anna Maryańska-Nadachowska

**Affiliations:** 1 Zoological Institute, Russian Academy of Sciences, 1 Universitetskaya Emb., St Petersburg 199034, Russia Zoological Institute, Russian Academy of Sciences St Petersburg Russia; 2 Institute of Systematics and Evolution of Animals, Polish Academy of Sciences, Sławkowska 17, 31-016 Kraków, Poland Institute of Systematics and Evolution of Animals, Polish Academy of Sciences Kraków Poland

**Keywords:** *18S rDNA*, completely inflated aedeagus, FISH, karyotype, male and female genitalia, morphology, Myanmar, new species, Oriental Region, Reduviidae, *Rhaphidosoma*, taxonomy, testes, (TTAGG)*_n_*

## Abstract

A new species, *Rhaphidosomapaganicum***sp. nov.** (Heteroptera: Reduviidae: Harpactorinae: Rhaphidosomatini), is described from the Dry Zone of Myanmar. It is the fifth species of *Rhaphidosoma* Amyot et Serville, 1843, known from the Oriental Region, and the first record of the genus for Myanmar and Indochina. The structure of the external and internal terminalia of the male and female is described and illustrated in detail. The completely inflated endosoma is described for the first time in reduviids. The complex structure of the ductus seminis is shown; it terminates with a voluminous seminal chamber which opens with a wide secondary gonopore and may be a place where spermatophores are formed. The new species is compared with all congeners from the Oriental Region and Western Asia. It is characterised by the absence of distinct tubercles on the abdominal tergites of the male, the presence only two long tubercles and small rounded ones on the abdominal tergites VII and VI, respectively, in the female, the presence of short fore wing vestiges which are completely hidden under longer fore wing vestiges, and other characters. In addition to the morphological description, an account is given of the male karyotype and the structure of testes of *Rh.paganicum***sp. nov.** and another species of Harpactorinae, *Polididusarmatissimus* Stål, 1859 (tribe Harpactorini). It was found that *Rh.paganicum***sp. nov.** has a karyotype comprising 12 pairs of autosomes and a multiple sex chromosome system (2n♂=24A+X_1_X_2_X_3_Y), whereas *P.armatissimus* has a karyotype comprising five pairs of autosomes and a simple sex chromosome system (2n♂=10A+XY). The males of these species were found to have seven and nine follicles per testis, respectively. FISH mapping of *18S* ribosomal DNA (major *rDNA*) revealed hybridisation signals on two of the four sex chromosomes (Y and one of the Xs) in *Rh.paganicum***sp. nov.** and on the largest pair of autosomes in *P.armatissimus*. The presence of the canonical “insect” (TTAGG)*_n_* telomeric repeat was detected in the chromosomes of both species. This is the first application of FISH in the tribe Raphidosomatini and in the genus *Polididus* Stål, 1858.

## ﻿Introduction

Harpactorinae are the largest subfamily of Reduviidae, comprising approximately 2,330 species and 320 genera in its worldwide distribution (Swanson 2020). The subfamily is composed of seven tribes: Apiomerini, Diaspidiini, Dicrotelini, Ectinoderini, Harpactorini, Rhaphidosomatini, and Tegeini (Weirauch et al. 2014; Zhang et al. 2015; Schuh and Weirauch 2020). The tribe Rhaphidosomatini Distant, 1904 has unclear circumscription and taxonomic status. Davis (1969) included five genera in this tribe: *Rhaphidosoma* Amyot et Serville, 1843, *Lopodytes* Stål, 1855, *Leptodema* Carlini, 1892, *Hoffmanocoris* China, 1940, and *Harrisocoris* Miller, 1959, while Maldonado Capriles (1990), only three genera: *Rhaphidosoma*, *Lopodytes*, and *Kibonotocoris* Miller, 1953. Swanson (2020), considering the taxonomic history of the tribe, concludes that it “became a ‘dumping ground’ or ‘trash bin taxon’ for any long, slender Old World harpactorine assassin bug”. The genera *Elongicoris* Hidaka et Miller, 1959, *Floresocoris* Miller, 1958, *Heterocorideus* Schouteden, 1952, *Pseudolopodes* Putshkov, 1985, *Pseudolopodytes* Schouteden, 1957, and *Vibertiola* Horváth, 1909 were not formally assigned to the tribe, but in the descriptions, they were compared with its representatives and share some of the distinctive characters of this tribe.

The genus *Rhaphidosoma* includes about 50 species distributed mainly in the Afrotropical Region. Twelve species are known in the Palaearctic, namely in North Africa and Western Asia, and only three species have so far been known in the Indian and Ceylonese subregions of the Oriental Region. In this article, a new species from the Indo-Chinese Subregion is described. As far as is known, species of this genus live in deserts and savannas in grass or low bushes (Miller 1956; Schuh and Weirauch 2020), and the find of a new species in the Dry Zone of Myanmar corresponds to these conditions.

To clarify the circumscription and status of the tribe Rhaphidosomatini, a detailed examination of the morphology of the listed taxa, with the involvement of new characters, including karyological ones, is necessary, and this work is a small step towards this goal. In particular, the completely inflated endosoma of a new *Rhaphidosoma* species, which has a complex structure and is rich in characters significant for taxonomy, is described for the first time for reduviids, and the structure of the female internal genitalia is also examined.

Our work was aimed also to describe the karyotype of a new species of *Rhaphidosoma* and search for the molecular composition of telomeres and localisation of *45S rDNA* loci in chromosomes of this species and another harpactorine species, *Polididusarmatissimus* Stål, 1859 (tribe Harpactorini), distributed in the Oriental and eastern Palaearctic regions. The standard karyotype of *P.armatissimus* was first studied by Banerjee (1958). Data on chromosomal FISH-mapping of repetitive DNAs are the first such data for the tribe Rhaphidosomatini in general. In addition, for both species, we studied the structure of the testes in terms of the number of testicular follicles they contain.

## ﻿Karyology of Harpactorinae

Previous original papers and revisions reported the chromosome complements of approximately 80 species (3% of the described ones) in about 40 genera (10% of the recognised ones) of Harpactorinae (Kpordugbe 1979; Ueshima 1979; Poggio et al. 2007; Kaur et al. 2012; Kaur and Kaur 2013; Bardella et al. 2014; Souza et al. 2014; Tiepo 2016; Grozeva et al. 2019). The vast majority of the studied species belong to the most species-rich tribe Harpactorini; however, several species have also been studied in the tribes Apiomerini (six species, two genera), Rhaphidosomatini (two species, two genera), and Dicrotelini (one species). Data available today suggest a high karyotypic uniformity of Harpactorinae, regarding the number of autosomes in their karyotypes. Despite the holokinetic nature of chromosomes, which is believed to facilitate karyotype evolution through chromosomal fusions and fissions (fragmentations), most harpactorine species have a diploid autosomal number of 24 (24A) as was concluded earlier by Poggio et al. (2007) based on an almost three times smaller data set. Species with a different number of autosomes (numbers 26, 22, 20, 18, 12, and 10 are presently known) are very rare, the second most common number, 22, being found mainly in the tribe Apiomerini (see Tiepo 2016). Another characteristic feature of harpactorines is multiple sex chromosome systems, with the number of X-chromosomes varying from one to five in different species (XY, X_1_X_2_Y, X_1_X_2_X_3_Y, X_1_X_2_X_3_X_4_Y, X_1_X_2_X_3_X_4_X_5_Y), while the system X_1_X_2_X_3_Y clearly predominates in the subfamily. Other mechanisms observed in other heteropterans (see Ueshima 1979; Poggio et al. 2007), such as X(0) and its multiple variant X_n_(0) as well as XY_n_ and neo-XY, have not been reported in harpactorines. A simple XY system seems to be characteristic of the tribe Apiomerini (all so far studied species of this small tribe have 2n=22A+XY) and is rarely found in the Harpactorini. It is generally argued that multiple systems usually arise from the simple systems XY or X(0) through chromosome fissions (Ueshima 1979; Poggio et al. 2013), although other mechanisms could also have taken place, e.g. non-disjunctions (Ueshima 1979) or duplications of the X-chromosome (Sadílek et al. 2019). It is also believed that autosomal translocation remnants can be fixed in population as extra sex chromosomes (Perez et al. 2004).

In recent years, knowledge of true bug cytogenetics has advanced significantly due to the use of modern techniques and approaches including chromosomal bandings, FISH, GISH, DNA content analysis, etc. (see for review Papeschi and Bressa 2007; Kuznetsova et al. 2021). This is also the case for the family Reduviidae (e.g. Bardella et al. 2014; Pita et al. 2016, 2021; Grozeva et al. 2019; Panzera et al. 2021). In particular, using fluorescence *in situ* hybridisation (FISH), it was shown that assassin bugs of the hematophagous subfamily Triatominae (Pita et al. 2016) and the subfamily Harpactorinae (Grozeva et al. 2019) display the “insect” telomere motif (TTAGG)_*n*_. This telomere sequence is considered as the ancestral one in the evolution of Heteroptera (Grozeva et al. 2011) and Hexapoda as a whole (Frydrychová et al. 2004; Vitková et al. 2005; Kuznetsova et al. 2020). It is important to emphasise that this motif was presumably lost by other families of Cimicomorpha; in any case, it could not be found in the families Cimicidae, Nabidae, Tingidae, and Miridae despite repeated attempts to detect it (Grozeva et al. 2011, 2019; Golub et al. 2018 and references therein). FISH with markers that identify specific chromosomes in a karyotype provides a powerful approach to studying general patterns and processes of evolution within a particular group (e.g. Sharakhov 2015). Repetitive DNAs are the main components of eukaryotic genome. FISH mapping of the major *rDNA* (tandemly arrayed *45S rDNA* repeating units encoding for the *28S*, *18S* and *5.8S rRNA*s), the most popular chromosomal landmark used in insect molecular cytogenetics, to reduviid chromosomes has been conducted multiple times. The largest amount of such data has been accumulated for the blood-feeding subfamily Triatominae (known as kissing bugs), in which as many as 92 species belonging to three of the five tribes, and ten of the 18 recognised genera have been studied to date (see Pita et al. 2021). As data accumulated on *rDNA* in triatomine karyotypes, it became clear that the conclusions reached from research on separate triatomine taxa apply to the subfamily as a whole. That is, triatomines are a significantly diverse subfamily in terms of the number and chromosomal location of the *45S rDNA* clusters. Triatomines have from one to four clusters per haploid genome and six distinct chromosomal location patterns (on one or two autosomes, on one, two or three sex chromosomes, on the X chromosome plus one to three autosomes). In triatomines, both number and chromosomal location of the *45S rDNA* clusters are species-specific, and the evolutionary mobility of *rDNA* clusters is supposed to be a driver of species diversification (Panzera et al. 2021; Pita et al. 2021).

The subfamily Harpactorinae is much less studied regarding chromosomal distribution of *rDNA*. Currently, data are available only for seven species in six genera (of the 2330 species described and 320 genera recognised; Swanson 2020), all these species belong to the most species-rich tribe Harpactorini (Bardella et al. 2014; Grozeva et al. 2019).

According to various authors, the tribe Rhaphidosomatini includes five (Davis 1969) down to three (Maldonado Capriles 1990) genera and approximately 90 species. Karyotype data are available for two genera, *Lopodytes* Stål, 1853 and *Rhaphidosoma* Amyot et Serville, 1843; all of them are obtained exclusively using routine methods of chromosomal staining. Different karyotypes were found in the two studied species, 2n=26(24A+XY) in *Rh.truncatum* Jeannel, 1914 (Kpordugbe 1979) and 2n=27(24A+X_1_X_2_Y) in *L.quadrispinosus* Villiers, 1948 (Louis and Kumar 1973). It was also shown that *L.quadrispinosus* has testes containing seven follicles (per testis) and ovaries containing seven ovarioles (per ovary); a similar structure of testes and ovaries was also discovered in two other studied species of Rhaphidosomatini, *Lopodytesarmatus* Villiers, 1948 and *Rhaphidosomaoccidentale* Jeannel, 1914 (Louis and Kumar 1973).

## ﻿Material and methods

The material for this study was collected by the first author during his expedition to Myanmar. The detailed data on the locality and the quantity of the material is provided in the section “Results and Discussion”: in the description of the new species (for *Rhaphidosomapaganicum* sp. nov.) and in the section “Chromosomal analysis” (for *Polididusarmatissimus*).

**Morphological studies.** The male and female terminalia were examined in wet preparations; the aedeagus was also examined in an entirely inflated condition in dry preparations made using the method of inflation by means of glass microcapillaries (Gapon 2001). The pygophores of several pinned specimens were removed and boiled for 2–3 min in 15–20% KOH solution. The abdomen of the female was boiled whole in a similar solution, whereupon the membrane between dorsal and ventral laterotergites was dissected, the abdominal dorsum was removed, and then the boiling of the abdomen without tergites was repeated. Gynatrial membranes and one of the aedeagi were stained with methylene blue. One male was boiled in alkali entirely to study the body sculpture and the endoskeleton. The phallobase and theca are described in repose.

The terminology for male genitalia partly follows Davis (1966); for endosoma, is based on the topographic principle and follows Konstantinov and Gapon (2005); for internal ectodermal genitalia of female, follows Scudder (1959), Štys (1961), Davis (1966), and Gapon (2007). In particular, although the term “gynatrial cone” was proposed by the first author for a morphologically separate part of the gynatrium, which accommodates the male vesica during copulation in Pentatomomorpha, this term is used here for a species that does not have a vesica, since the part of its gynatrium, into which the common oviduct opens, is also morphologically separated and has a distinct structure similar to that of Pentatomomorpha. Membranous elements of the sculpture of the endosoma, which are wider or subequal to their length, are here named tubercles, those that are longer than their width are named lobes.

All measurements are given in millimetres.

The holotype and paratypes of the new species are stored at the Zoological Institute of the Russian Academy of Sciences, St Petersburg, Russia (ZISP).

**Chromosome studies.** One male of the new species and eight males of *Polididusarmatissimus* were studied using conventional chromosome staining and FISH with *18S rDNA* and (TTAGG)*_n_* probes.

**Fixation and slide preparation.** Whole individuals were fixed immediately after collecting in 3:1 (ethanol: acetic acid) fixative. Chromosome preparations were made from the male gonads. The testes were extracted from the abdomen; testicular follicles were separated from each other in order to determine their number and shape, placed on a slide in a drop of 45% acetic acid, covered with a coverslip, and squashed by gently pressing it. No more than one or two follicles were placed on each slide. The coverslip was removed with a razor blade after freezing with dry ice, and the slide was then dehydrated in fresh fixative (3:1) and air dried.

**Routine staining.** For this staining, we followed the Schiff-Giemsa method (Grozeva and Nokkala 1996), with 30 min Schiff staining and 40 min Giemsa staining.

**Fluorescence in situ hybridisation (FISH).** Probes for *18S rDNA* and (TTAGG)_*n*_ were prepared and two-colour FISH was carried out as described by Grozeva et al. (2019); for primer information, see Grozeva et al. (2011). The telomere probe was amplified by PCR and labelled with rhodamine-5-dUTP (GeneCraft, Köln, Germany). An initial denaturation period of 3 min at 94 °C was followed by 30 cycles of 45 s at 94 °C, annealing for 30 s at 50 °C and extension for 50 s at 72 °C, with a final extension step of 3 min at 72 °C. The *18S rDNA* probe was amplified by PCR and labelled with biotin-11-dUTP (Fermentas, Vilnius, Lithuania) using genomic DNA of the firebug, *Pyrrhocorisapterus* (Linnaeus, 1758). An initial denaturation period of 3 min at 94 °C was followed by 33 cycles of 30 s at 94 °C, annealing for 30 s at 50 °C and extension for 1.5 min at 72 °C, with a final extension step of 3 min at 72 °C. The chromosome preparations were treated with 100 μg/ml RNase A and 5 mg/ml pepsin solution to remove excess RNA and proteins. Chromosomes were denatured in the hybridisation mixture containing labelled *18S rDNA* and (TTAGG)*_n_* probes (80–100 ng per slide) with an addition of salmon sperm blocking reagent and then hybridised for 42 h at 37 °C. *18S rDNA* probes were detected with NeutrAvidin-Fluorescein conjugate (Invitrogen, Karlsbad, CA, USA). The chromosomes were mounted in an antifade medium (ProLong Gold antifade reagent containing 40,6-diamidino-2-phenylindole (DAPI) (Invitrogen) and covered with a glass coverslip. All nine males examined in both species showed a large number of dividing meiotic cells, then, dozen cells were scanned. We had unfortunately no females to study, and therefore the female karyotypes cannot be mentioned.

**Microscopy and imaging.** The routinely stained preparations were analysed using an Olympus BX 51 light microscope with an Olympus C-35 AD-4 camera. FISH images were taken using a Leica DM 6000 B microscope with a 100×objective, Leica DFC 345 FX camera, and Leica Application Suite 3.7 software with an Image Overlay module (Leica Microsystems, Wetzlar, Germany). The filter sets applied were A, L5 and N21 (Leica Microsystems). The specimens, from which the chromosome preparations have been obtained, are stored at the Zoological Institute RAS (St Petersburg, Russia).

## ﻿Results and discussion

### ﻿Family Reduviidae

#### Subfamily Harpactorinae

**Tribe Rhaphidosomatini**

##### Genus *Rhaphidosoma* Amyot et Serville, 1843

###### ﻿ Rhaphidosoma paganicum

Taxon classificationAnimaliaHemipteraReduviidae

Gapon sp. nov.

94AC831B-7412-5F6D-8312-770367383DE2

http://zoobank.org/32F81B06-1A3A-4111-A923-48E7F1A861BA

Figs 1 , 2 , 3 , 4 , 5 , 6 , 7 , 8 , 9

####### Material examined.

***Holotype*.** Male (glued to rectangular piece of card), **Myanmar**, **Mandalay Region**, nr Nyaung-U Town, 21°10'47.2"N, 94°53'37.9"E, 31.X.2019, D.A. Gapon leg. (ZISP).

***Paratypes*.** Same data as for holotype, 5 males, 1 female mounted as holotype, 3 males in ethanol, 1 male in fixative, with a series of karyological preparations on slides (ZISP).

####### Description.

Body strongly elongated, rod-shaped, with subparallel lateral margins, slightly widened at level of thoracic segments. Antennae and legs long and thin. Both sexes with vestigial fore and hind wings.

***Colouration and integument*.** Body dark reddish-brown, often with blackish sides; ventral surface of abdomen with a more or less distinct median yellowish stripe; tarsi and last two segments of antennae yellowish brown; claws and apex of last segment of rostrum black. Body entirely, except for last two segments of rostrum, covered with dense, adpressed, whitish scale-like setae; they rather long on head, slightly shorter on thorax and abdomen, and very short and sparse on antennae and legs. On abdomen of female, they are shorter than on that of male, therefore looking less dense. Head ventrobasally with very long and dense erect setae; similar long setae, directed anteriorly, located at anterior angles of pronotum; thoracic sternites in their anterior and posterior parts near coxal cavities with dense, rather long, raised setae.

Some setae all over body, including legs and antennae, semierect, located on sparse, rounded, minute setiferous tubercles, those being visible only on wet preparations and hidden by setae on dry specimens. Such tubercles on dorsal and lateral surfaces of postocular part of head larger (looking like granules on dry specimens), bearing relatively long setae. Semierect setae on preocular part of head slightly shorter, becoming longer and denser on dorsal surface of clypeus; several longer setae located laterally anterior to eyes and on antennal tubercles. Ventral surfaces of head and thorax with less long semi­erect setae, dorsal surface of thorax and whole abdomen with short semierect setae; first segment of each antenna in basal part with semierect setae slightly shorter than thickness of the segment and increasing in length towards its apex; second segment with setae not exceeding its thickness and also with sparse, longer setae; third and fourth segments with very short, semierect setae. Semierect setae on coxae, trochanters and femora rather short, longer, denser on tibiae and becoming longer towards apices of tibia.

V-shaped spot on dorsal surface of preocular part of head, longitudinal stripes on sides of ventral surface of head, a thin medial stripe on ventral surface of abdomen, lateral irregular stripes and extreme margins of abdominal ventrites with very sparse, almost absent pubescence. Rostrum shining, with sparse, very short, semierect setae, only its first visible segment with adpressed pubescence and several longer, semierect setae. Small, round, dark, shining, slightly depressed scarlike marks located in pairs on abdominal tergites: approximately in middle of combined tergites I–III and before middle of each subsequent tergite; the same markings, longitudinal anterior and rounded posterior, located at anterior angle of each abdominal ventrite.

Simple scale-like setae and cuticle between them covered with a white waxy coating, that being especially abundant and thick on ventral surface of body.

**Figure 1.**
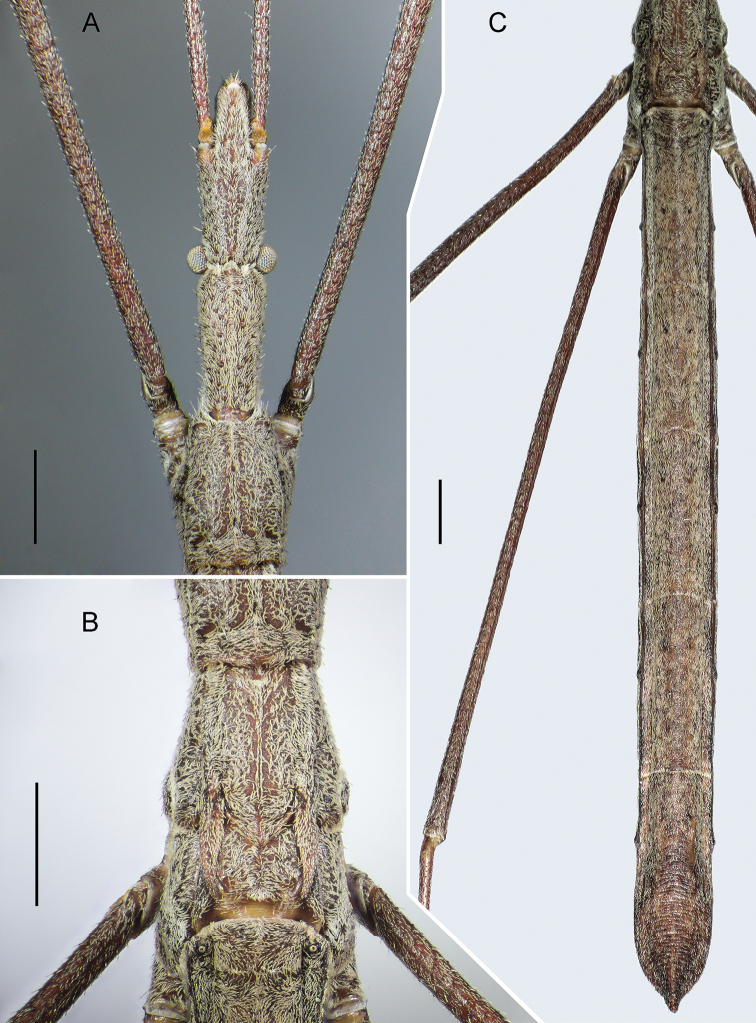
*Rhaphidosomapaganicum* sp. nov., holotype in dorsal view **A** head and prothorax **B** meso- and metathorax **C** abdomen. Scale bars: 1 mm.

**Figure 2.**
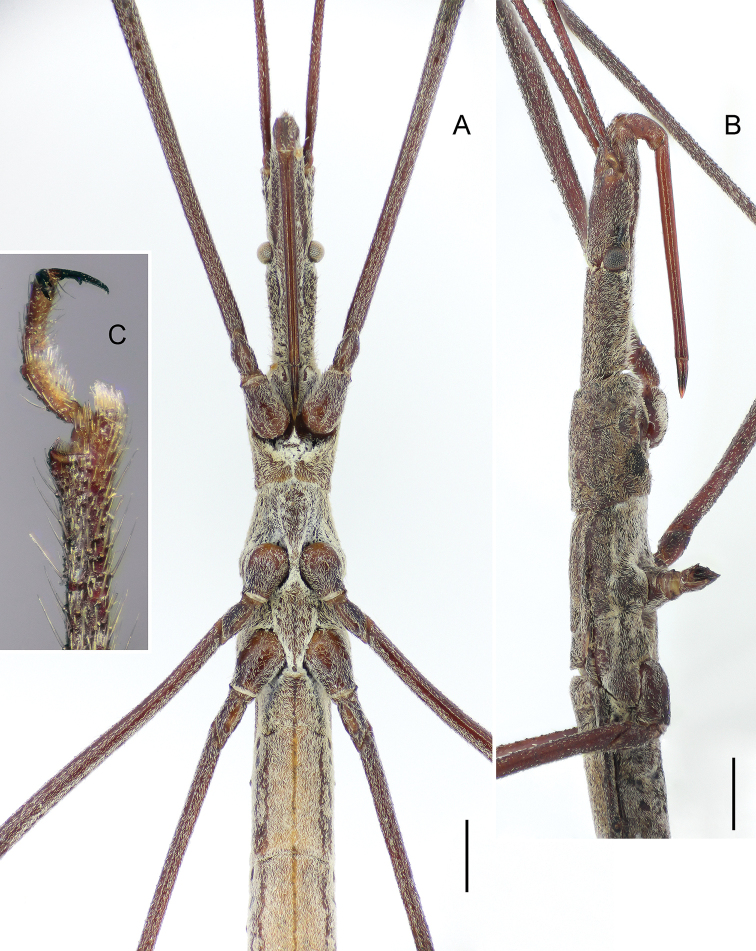
*Rhaphidosomapaganicum* sp. nov. **A** anterior part of body in ventral view **B** anterior part of body in right lateral view (right fore and middle legs omitted) **C** extreme apex of fore leg in anterior view. Holotype (**A**) and paratype (**B, C**). Scale bars: 1 mm.

***Head*** (Figs 1, 2) long, linear, with deep transverse interocular sulcus. Postocular part of head slightly widened laterally just behind eyes, slightly narrower than anteocular part at level of antenniferous tubercles; its anterior margin with a small medial triangular projection directed anteriad. The part of head anterior to sulcus faintly wide­ning towards antenniferous tubercles. Head dorsally and ventrally almost flat, only slightly convex just posterior to clypeus base. Eyes moderate, hemispherical. Ocelli absent. Clypeus rather wide, conical, slightly flattened laterally, obtuse at apex, without sharp spine anteriorly. Antenniferous tubercles large, far removed from eyes, faintly diverging, completely visible from above. Segments II–IV of antennae thinner than segment I, gradually thinning distally. Maxillary plate triangular, flat. Gena convex. Rostrum straight, reaching middle of fore coxae; its first visible segment significantly not reaching base of antenna, second segment reaching posterior margin of head, but not protruding beyond it; third segment slightly shorter than first one. Labrum short, about three-fifths of first rostral segment.

**Figure 3.**
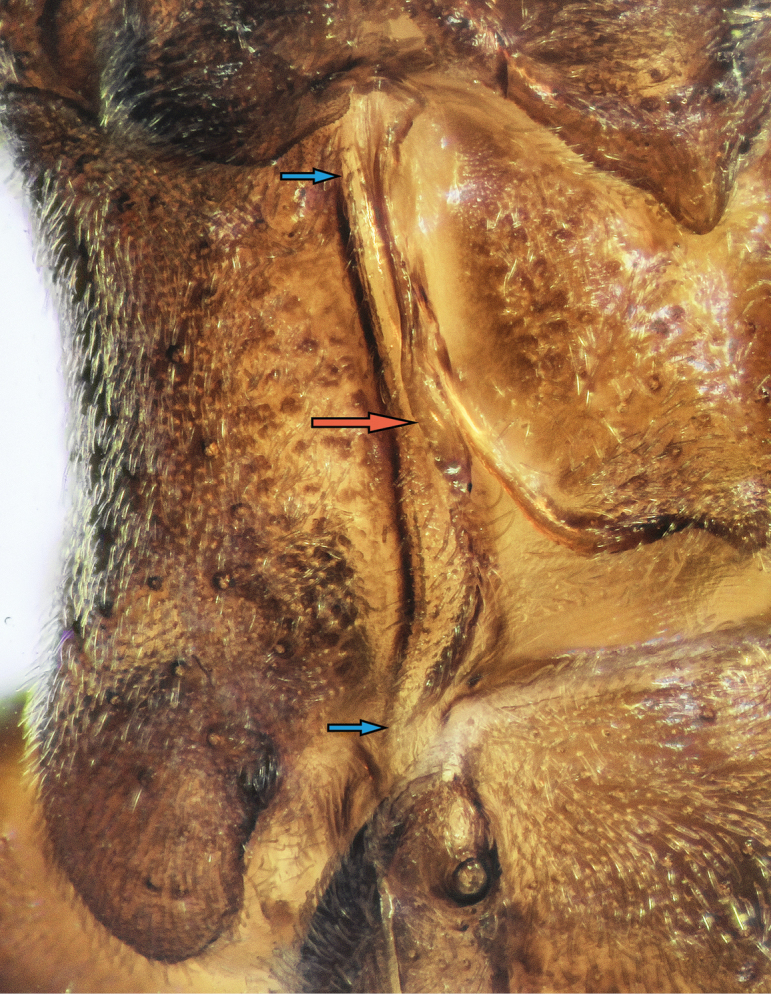
*Rhaphidosomapaganicum* sp. nov., metanotum in left dorsolateral view at a wet preparation, under high magnification. Vestige of the left forewing is removed. Red arrow indicates a vestige of the hind wing, and blue arrows, lateral carina of the metanotum.

***Thorax*** (Figs 1–3). Anterior margin of pronotum deeply notched; lateral margins without carinae, almost parallel in posterior half, converging anteriorly in anterior half; posterior margin slightly concave laterally, convex, raised medially. Anterior angles slightly elongated, angularly rounded. Anterior lobe of the pronotum long, slightly convex, with a thin medial sulcus posteriorly; its posterior margin looking like two letters W, i.e. with four triangular projections rising above posterior lobe of pronotum. The latter short, rim-like; its medial area flat, with extremely smoothed medial and lateral carinae converging posteriorly; lateral areas deeply depressed. Pleural areas of prothorax relatively weakly convex, but clearly visible from above. Each epimeron continuing ventrally like a long plate; together they enclose the posterior part of sternite and touch with their inner margins. Rather large, rounded spiracle located on a plate surrounded by membrane under each epimeron and not visible from outside.

Labial sulcus wedge-shaped, tapering posteriorly, with thinnest transverse stridulation ribs, bounded by rounded keels, tapering and converging posteriorly. Coxal cavities located near anterior margin of prothorax, closed posteriorly and open anteriorly, separated from each other by a very narrow, sharp carina. Fore coxae contiguous. Each coxal cavity posteriorly with rather large, rounded fossa, corresponding to large internal hook-shaped apodeme lying in transverse plane, having flattened apex directed medially and ventrally.

Mesothorax wider than prothorax, trapezoidal, its pleural areas wide, with margins converging anteriorly in dorsal view. Mesonotum narrower than pronotum, its lateral margins almost parallel, slightly convex in middle, slightly concave anteriorly and posteriorly. Disc slightly convex, without a distinct medial sulcus or carina, laterally bounded along its entire length by a pair of wide flattened carinae, those being strongly smoothed anteriorly and passing into vestiges of fore wings posteriorly. The latter narrow, elongate-triangular, incumbent on metanotum, reaching its posterior margin, slightly curved medially before pointed apices. Scutellum distinct, slightly shorter than its width, convex, fused with mesonotum without distinct suture, with sharp apex and posterior margin bordered by rounded carina thickening towards middle. Small, rounded spiracle located under convex posterior margin of epimeron near dorsal margin of pleurite.

Middle coxal cavities open anteriorly, separated each from other by wider than in prosternum, flat, raised carina. Middle coxae slightly more widely spaced than anterior ones.

Metathorax short, slightly wider than mesothorax, its pleural areas wide in dorsal view, with lateral margins subparallel in anterior part and diverging posteriorly in posterior part. Metanotum slightly wider than mesonotum; its disc rather convex, without a medial sulcus or carina, laterally bounded by lateral carinae. They narrow, subparallel, hidden by vestiges of fore wings in anterior half and, in posterior half, wide, flat, tapering towards posterior ends, reaching posterior margin of metathorax and diverging. Base of each hind wing vestige looking like a narrow longitudinal carina, medially adjacent to anterior half of each lateral carina of metanotum (Fig. 3). Distal part of each vestige shaped as narrow platelike flap with narrowly rounded apex. Vestiges of hind wings completely hidden under those of fore wings. Posterior margin of metanotum tapering trapezoidally posteriorly, roundly convex laterally, finely concave in middle, with small triangular median projection, and framed by thin rounded carina starting from base of posterior part of each hind wing vestige. Posterior part of tergite flat, smooth. Small rounded spiracle located near posterior margin of pleurite in its dorsal part at level of posterior end of metanotal lateral carina.

Hind coxal cavities closed anteriorly, open posteriorly. Hind coxae wider moved apart than middle ones. Space between middle and hind coxal cavities monolithic, rhomboid, elongated posteriorly, with almost flat, barely depressed surface and thin medial sulcus. Ostiole of metathoracic scent gland and evaporatorium absent. Epimeron shaped as large, triangular, convex plate with rounded posterior margin.

***Note*.** It seems that in the description of some species of *Rhaphidosoma*, posterior ends of lateral carinae of the mesonotum are confused with vestiges of the hind wings, which are actually absent in these species. Genuine vestiges of the fore and hind wings are described in this article, and the representation of this character in the genus requires clarification.

***Legs*.** Coxae of all legs longitudinal, swollen; femora and tibiae evenly slender, without any denticles. Anteromedial surface of each fore tibiae subapically with distinct comb (Fig. 2C). Tarsi three-segmented, with a very small first segment. Claws long, thin, slightly curved, with a long thin denticle before middle.

**Figure 4.**
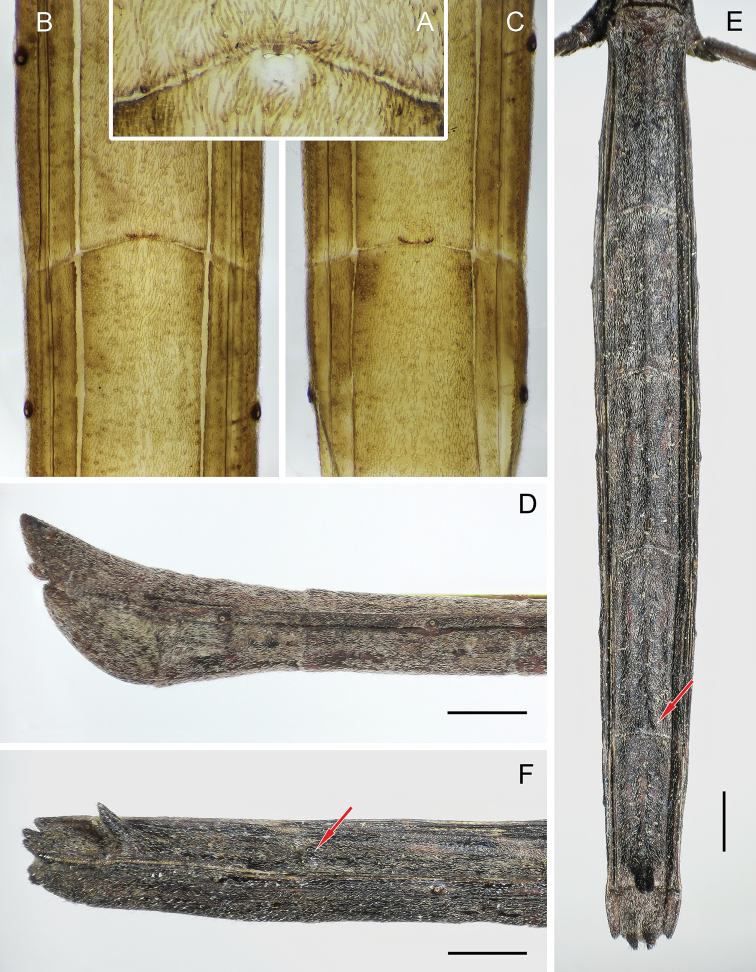
*Rhaphidosomapaganicum* sp. nov. **A** boundary between tergites IV and V, and opening of abdominal scent gland in male (paratype) **B** boundary between tergites V and VI in male (paratype) **C** boundary between tergites VI and VII in male (paratype) **D** apex of abdomen of male (paratype) in right lateral view **E** abdomen of female in dorsal view **F** apex of abdomen of female in right dorsolateral view. Wet preparations (**A–C**) and dry specimens (**D–F**). Red arrows indicate small tubercles on the posterior margin of tergite VI in female. Scale bars: 1 mm.

***Abdomen*** (Figs 1, 2, 4) with lateral margins parallel in male and slightly convex towards middle in female. Tergites I, II and III seamlessly merged; on preparations cleared in alkali, border between tergites II and III barely discernible as area of ​​weaker sclerotisation. Inner surface of combined tergite I–III at border of short I and longer II tergites with large fragma looking like two contiguous, wide, rather high crests with semicircular ventral margins, anteriorly concave surfaces, and rather long apodeme at each of lateral ends. Tergites evenly, not strongly convex, only base of combined tergite I–III rather strongly elevated. Posterior margins of tergites from III to V rather deeply concave; posterior margin of tergite VI weakly and smoothly concave. Male and female with only two dorsal abdominal scent glands located at anterior margin of tergites IV and V, with openings shaped like an eight (Fig. 4A).

Ventrites rather strongly convex; boundaries between ventrites II and III on inner side with two slightly spaced, concave, transverse cristae having a cupped shape, concave anteriorly. Posterior margins of ventrites from II to IV rather deeply concave and less concave in subsequent ventrites.

Connexivum separated dorsally by a rather deep depression. Ventral connexival suture present. Dorsal and ventral laterotergites flat, almost vertical.

Spiracles located on small tubercles. First pair of spiracles located dorsally close to anterior margins of corresponding laterotergites; their tubercles directed slightly posteriad (Fig. 1). Spiracles of second pair small, lying very close to spiracles of first pair; third pair of spiracles larger, located slightly behind middle of combined tergite I–III and before middle of ventrite III; other pairs of spiracles located slightly anterior to middle of corresponding tergite and ventrite.

Posterior margin of median tergite VI in male with low, smoothed, transverse medial elevation only with a row of 5–6 setiferous tubercles at posterior margin (Fig. 4C); such elevation on tergite V even less distinct, also with a row of setiferous tubercles (Fig. 4B). Posterior margins of previous tergites only slightly convex in middle. Tergite VII posteriorly weakly widened, with smoothly rounded lateral margins in dorsal view. Posterior margin of this tergite transversely wrinkled, elongated, pointed, strongly raised, protruding far beyond posterior margin of pygophore, carinate at extreme apex (Figs 1C and 4D). Laterotergites VII terminated behind middle of the median tergite, fused with it in dorsal view and, in lateral view, gradually narrowing posteriorly and smoothly passing into thin carina bordering posterior margin of tergite VII. Segment VIII completely retracted into previous segment; its ventral sclerotised part represented by rather long semicircle with oblique anterior margin.

Tergites in female with three weak longitudinal carinae disappearing anteriorly. Posterior margin of tergite VII medially with two long, digitiform, contiguous throughout tubercles, those being located on rather high common elevation and directed dorsoposteriorly (Figs 4E, F and 8A). Posterior margin of tergite VI weakly raised in middle, with two small, rounded tubercles contiguous anteriorly and slightly spaced posteriorly (Fig. 8B). Previous tergites without any distinct tubercles. Tergite VIII short, with medial sulcus and two rather long, slightly spaced conical processes of posterior margin, those being directed posteriorly and slightly dorsally (Fig. 8C). Connexival membrane between dorsal and ventral laterotergites extensive, with multiple thin and one large longitudinal folds (Fig. 8A). Dorsal and ventral laterotergites of segment VIII fused with each other; dorsal ones fused with the median tergite; ventral laterotergite with small spiracle in anterior part. Posterior margin of ventrite VII with small medial triangular projection.

**Figure 5.**
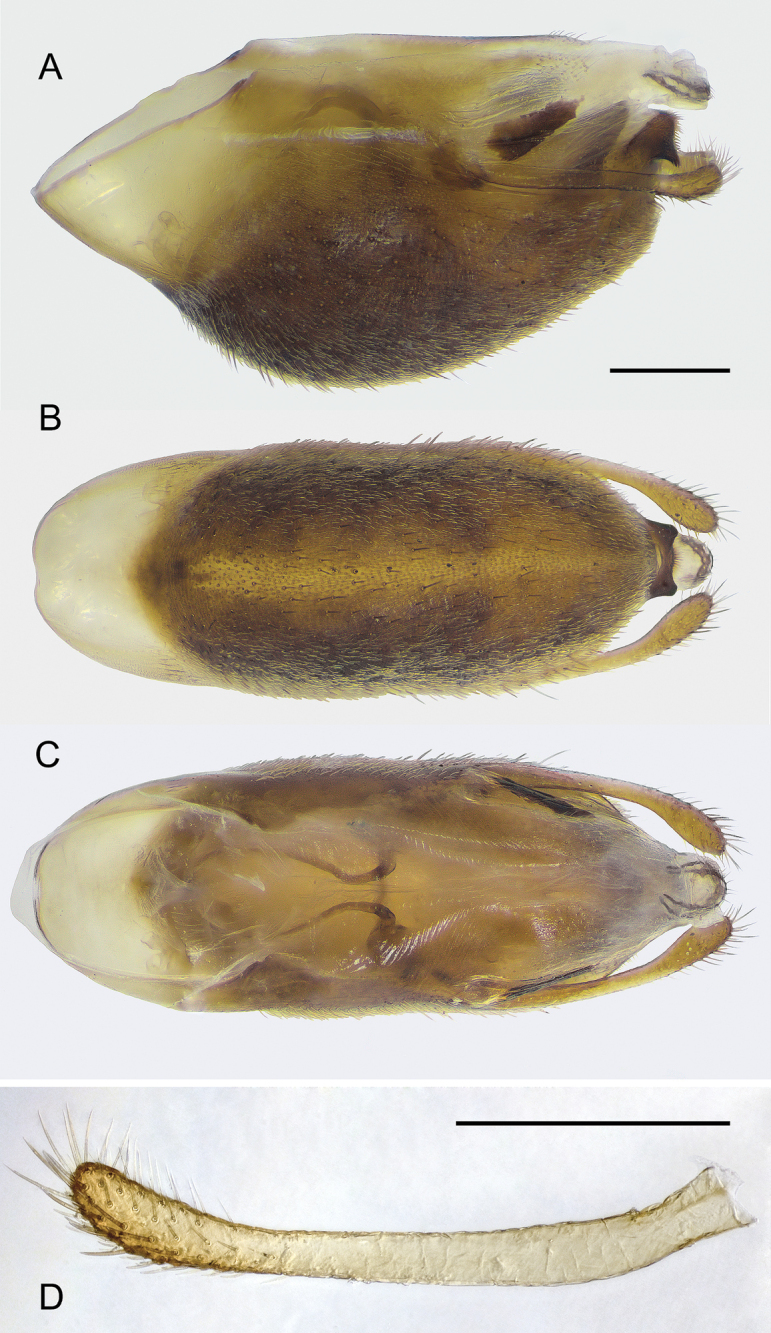
*Rhaphidosomapaganicum* sp. nov., pygophore (**A–C**) and right paramere (**D**) at wet preparations (paratype) **A** left lateral view **B** ventral view (ventral and lateral branches of basolateral lobes of endosoma are slightly protruding beyond theca under the action of osmotic pressure **C** dorsal view **D** right lateral view. Scale bars: 0.5 mm.

***Pygophore*** (Fig. 5A–C) 2.5 times as long as wide. Its dorsal wall straight, ventral wall strongly and rather smoothly convex before middle, lateral walls almost parallel. Basal foramen large, longitudinally oval, oblique. Lateral and ventral walls sclerotised (ventral one stronger), covered with dense, appressed scale-like setae, as well as sparser, thickened, semierect setae. At extreme base of pygophore, these walls weaker sclerotised, without pubescence. Ventral wall with light medial stripe. Lateral walls membranous dorsodistally, each with triangular, strongly sclerotised isolated area above base of paramere and anterior to base of proctiger. Paramere attached at anterioventral margin of this membranous area. Dorsal wall of pygophore membranous, deeply folded along midline, without visible border passing into large, cone-shaped, membranous proctiger posteriorly hanging over apex of pygophore. Dorsal and ventral valves of proctiger reinforced with thin horseshoe-shaped sclerites. Laterally, dorsal wall with two longitudinal, weakly sclerotised areas anteriorly covered with thin oblique wrinkles and, posteriorly, with thin, semierect setae. Apex of pygophore rounded in ventral view; medial process highly sclerotised, shaped as wide base with two wide denticles directed ventroposteriad. Genital opening located terminally, bounded by sclerotised apex of pygophore, membranous ventral wall of proctiger and membranous portions of lateral walls of pygophore; it small in repose, but able to stretch strongly due to elasticity of the membranes.

***Paramere*** (Fig. 5D) long and narrow. Corpus cylindrical, without any projections, slightly curved at base, almost straight in rest part. Hypophysis slightly widened and slightly flattened laterally, slightly curved dorsally and medially; its ventral margin more convex than dorsal one; apex rounded; lateral surface, dorsal and ventral margins covered with rather long setae. Parameres located horizontally along lateral walls of pygophore, posteriorly protruding far beyond its apex.

**Figure 6.**
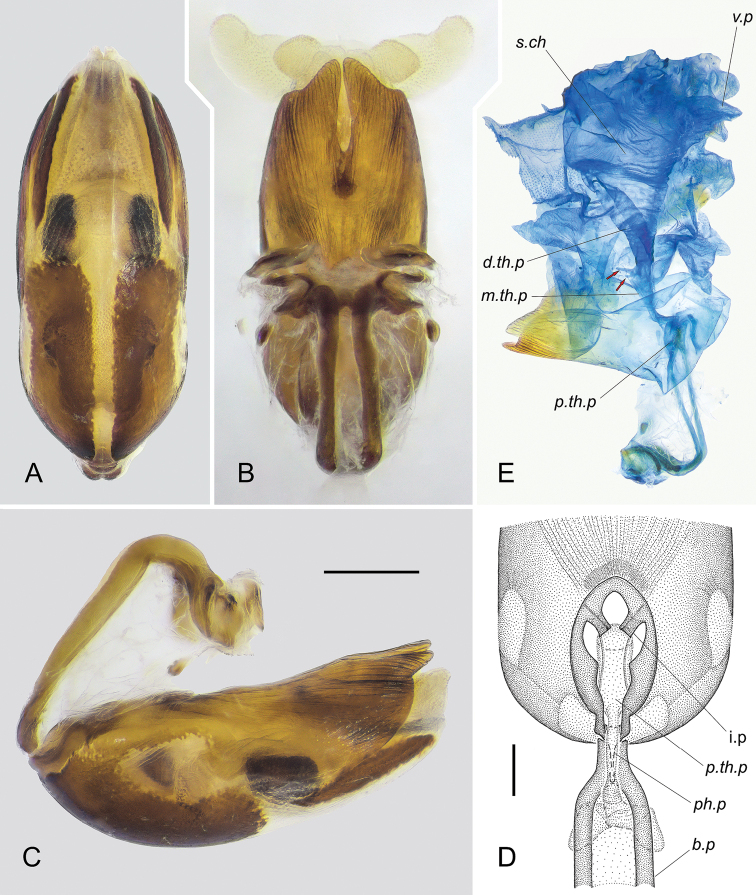
*Rhaphidosomapaganicum* sp. nov., aedeagus (paratype) at wet preparations **A–C** theca and phallobase in ventral (**A**), dorsal (**B**) and left lateral (**C**) view **D** basal foramen of theca in anteriodorsal view (phallobase is strongly unbent anteriad, membrane connecting the basal plates and theca is removed and not shown) **E** ductus seminis in dissected aedeagus, right lateral view. Abbreviations: *b.p* – basal plate of phallobase; *i.p* – inner process of thecal basal foramen; *ph.p* – phallobasal part of ductus seminis; *d.th.p* – distal thecal part of ductus seminis; *m.th.p* – middle thecal part of ductus seminis; *p.th.p* – proximal thecal part of ductus seminis; *s.ch* – seminal chamber; *v.p* – ventral pouch of seminal chamber. Red arrows indicate cords attached to the middle thecal part of ductus seminis. Scale bars: 0.5 mm.

***Aedeagus*.** Basal plates of phallobase (Fig. 6B, C) very long, almost parallel, slightly widening posteriorly in lateral view; their posterior ends C-shaped, curved ventrally and diverging, extreme ends widened, with spoon-shaped depression. Suspensory apo­demes short, attached laterally to almost extreme apices of posterior ends of basal plates. Dorsal apodemes very short, attached to ends of basal plates medially and subterminally. Capitate processes large, broadly oval, with irregular margin and extremely short stalk. Plate bridge narrow and rather short. Pedicel short, ductifer absent.

Theca (Fig. 6A–C) cylindrical, slightly flattened dorsoventrally, with almost parallel lateral walls, tapering anteriorly and posteriorly, narrowly rounded at anterior end. Dorsal wall of theca entirely sclerotised (“dorsal phallothecal sclerite”), except for two small weakly sclerotised windows located on either side of place of attachment of phallobase. Basal foramen of theca (Fig. 6D) rather large, located at base of its dorsal wall, longitudinal, bordered by flat, rather wide edging with distinct outer margins fused with membrane connecting theca and basal plates of phallobase. Inner margins of this plate in middle with a pair of triangular projections, posteriorly with a pair of large rectangular inner processes directed slightly into cavity of theca; anterior angle of each of these processes and margin of basal foramen in its anterior part connected by very thin transparent cord.

Ventral wall of theca in basal half with two longitudinal, almost rectangular areas of strong sclerotisation, those being separated by rather wide membranous interval. At extreme base, these areas fused with sclerotisation of dorsal wall of theca, extending onto its lateral sides. In middle of theca length, dorsal sclerotisation extending even further to sides and almost touching lateroapical angles of strongly sclerotised parts of ventral wall. Lateral walls of theca in its basal half between described areas of sclerotisation remain membranous. Apical half of ventral wall of theca membranous, with two narrow, highly sclerotised stripes laterally, those being tapering anteriorly and posteriorly. Extreme thecal apex represented by two lobes tapering apically. Dorsal lobe completely sclerotised, with deep, narrow medial notch and external surface striated with dense longitudinal wrinkles. Ventral lobe membranous, trapezoidally rounded apically, with lateral margins reinforced with narrow stripes of strong sclerotisation described above, basally extending under lateral margins of dorsal lobe.

Short unpaired ectodermal vas deferens entering cavity of aedeagus near place of fusion of basal plates (near posterior end of pedicel). Ductus seminis morphologically subdivided into five parts (Fig. 6E): (1) phallobasal part short, narrow, extending into cavity of theca at anterior margin of its basal foramen; (2) proximal thecal part sharply widening at base, wide, running along plane of basal foramen and connecting to inner processes of the latter; (3) middle thecal part curved at right angles to the previous part, narrow at base and gradually widening distally; (4) distal thecal part widening very strongly, funnel-shaped and passing into (5) voluminous seminal chamber opening by very wide secondary gonopore. Ventral wall of seminal chamber with large triangular pouch jutting into cavity of endosoma. At distal end of middle thecal part of ductus seminis, two thin cords (indicated by red arrows in Fig. 6D) attached to its dorsal wall (in another specimen, they are fused with each other into a ring-shaped structure; their functional significance is unclear).

**Figure 7.**
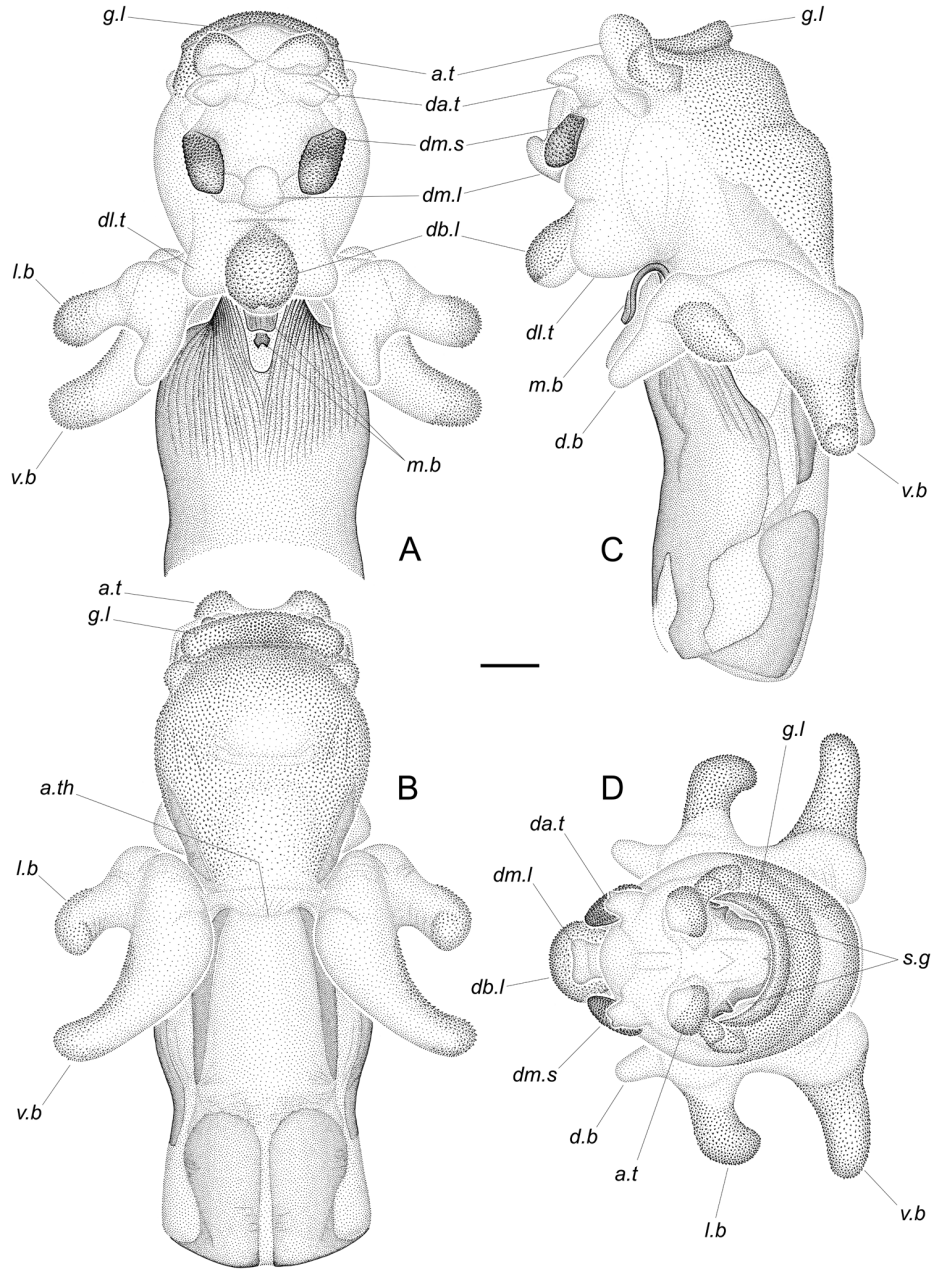
*Rhaphidosomapaganicum* sp. nov., completely inflated aedeagus (paratype) at dry preparations **A** dorsal view **B** ventral view **C** left lateral view **D** caudal view. Phallobase not shown. Abbreviations: *a.t* – apical tubercle; *a.th* – apical margin of theca; *d.b* – dorsal branch of basolateral lobe; *da.t* – dorsoapical tubercle; *db.l* – dorsobasal lobe; *db.t* – dorsolateral tubercle; *dm.l* – dorsomesial lobe; *dm.s* – dorsomesial sclerite; *g.l* – gonoporal lip; *l.b* – lateral branch of basolateral lobe; *m.b* – medial band of the basal part of endosoma; *s.g* – secondary gonopore; *v.b* – ventral branch of basolateral lobe. Scale bar: 0.5 mm.

Endosoma (Fig. 7) subdivided into basal and apical parts. Basal part with two large basolateral lobes, each having three branches. Ventral branches long, swollen at base, narrowed in distal part, directed ventrolaterally, with extreme apices rounded, slightly curved dorsoposteriorly; dorsal branches short, tapering towards pointed apices, directed dorsoanteriorlly and slightly diverging; lateral branches located strongly close to dorsal ones on common with them, slightly swollen base, slightly longer than dorsal branches, directed laterally and slightly anteriorly, widened before rounded apices, those being C-shaped, rather strongly curved ventrally. Lateral and posterior surfaces of ventral and lateral branches densely covered with finest microspines directed to apex of corresponding branch. Dorsal wall of basal part of endosoma with narrow, highly sclerotised medial band; distally it passes into a long plate, that being C-shaped, sharply curved anteriad (only on completely inflated endosoma; in repose, this plate almost straight, adjacent to dorsal wall of endosoma), slightly widening distally, with rounded lateroapical angles and notched apical margin. Ventral wall of basal part of endosoma extremely short, delimited from the apical part by transverse fold.

Apical part of endosoma large, obovoid. Its base convex on dorsal side, bearing unpaired dorsobasal lobe and paired dorsobasal tubercles located laterally and slightly more basal than base of the latter. Dorsobasal lobe short, rounded, slightly tapering towards base, directed dorsoanteriorlly. Posterior surface of this lobe with sharply outlined field of microsculpture represented by dense, larger than on basolateral lobes, rather strongly sclerotised, tangentially flattened microspines decreasing towards base of lobe. Apex of lobe with two tiny contiguous tubercles. Dorsobasal tubercles shorter than dorsobasal lobe, tapering broadly to rounded apices directed anterolaterally; surfaces of tubercles without microspines. Distal part of dorsal wall of endosomal apical part smoothly convex, with unpaired dorsomesial lobe, paired dorsomesial sclerites lying on either side of the latter, and with paired membranous dorsoapical tubercles located even more distally. Dorsomesial lobe small, narrowed basally, widening distally, directed dorsoanteriorlly, slightly curved posteriad, without microsculpture. Dorsomesial sclerites rather large, diamond, with rounded angles, posteriorly fused with wall of endosoma, with anterior ends slightly elevated above the wall. Dorsal surface of each sclerite convex, densely and very finely granulated. Dorsoapical tubercles widely spaced, small, conical, with pointed apices directed dorsally, without microsculpture.

Apex of apical part of endosoma broadly dome-shaped, with paired membranous apical tubercles on dorsal side and transverse membranous protuberance on ventral side. Apical tubercles rather large, almost hemispherical, spaced apart. Membranous protuberance crescent, wide, rounded in cross section, rather thick in middle, thinning dorsally towards ends, bordering secondary gonopore ventrally, and here named gonoporal lip. Secondary gonopore looking like a wide gap between gonoporal lip and apex of endosoma, very short in middle, widening laterally; its dorsal margin at each end with a small conical membranous tubercle and very densely covered with finest microsculpture, resulting in it looking dark brown. Ventral half of each apical tubercle, lateral portions of endosomal apex, and entire gonoporal lip densely covered with microspines becoming denser towards middle of the latter.

Ventral wall of apical part of endosoma flat in proximal part and convex distally, with shallow transversal depression behind middle, entirely covered with finest microspines (those being smaller than at apex of endosoma). Distally, this area of microspines somewhat continuing onto lateral walls and, basally on each side, edged by oblique, very weakly sclerotised band.

***Notes on functional morphology, dissection and terminology of the aedeagus*.** In repose, the basal part of endosoma is simply retracted into the theca, while the large apical part is turned inside out like a glove, and when straightened, it should turn back through a relatively small opening at the basal part of endosoma. This mode of folding the endosoma greatly complicates making preparations of completely inflated aedeagi. Perhaps for that reason and because of the high water pressure required in this case, the only completely inflated preparation that the first author (D.G.) obtained well, burst in two places, on the dorsal ends of the gonoporal lip, and these places were reconstructed in the drawings. It should also be noted that all the microspines in the drawings are shown slightly larger than they are, since D.G. did not have the technical ability to draw them very thin.

Perhaps the sclerotised medial band of the basal part of endosoma corresponds to the merged “struts” of some other reduviids, although D.G. does not quite comprehend what “struts” are as described by Davis (1966); he points out that in “Rhaphidosominae”, “the struts are short, widely separated, and attached to the proximal [sic!] edge of the dorsal phallothecal sclerite”, but D.G. did not find such structures in the aedeagus of the species described, and he believes that no endosomal structures can attach to the proximal part of the theca.

It is hard not to associate the complex structure of the ductus seminis, the extreme distal part of which is represented by the extensive seminal chamber with the large pouch and opens by the wide secondary gonopore possessing soft margins, with the presence of spermatophores in insemination in reduviids (about this see e.g. Ambrose and Vennison 1990). It can be assumed that the seminal chamber is the place where spermatophores form or complete their formation.

**Figure 8.**
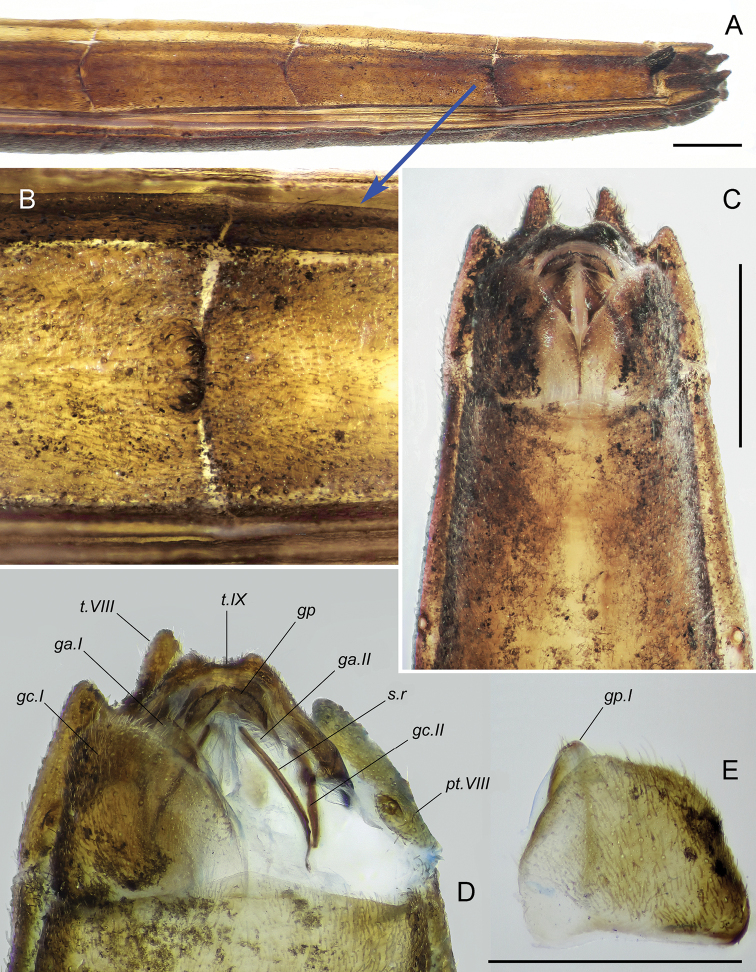
*Rhaphidosomapaganicum* sp. nov., details of the structure of female abdomen, at wet preparations **A** apical part of abdomen in left dorsolateral view **B** boundary between tergites VI and VII under high magnification **C** extreme apex of abdomen in ventral view **D** terminalia in ventral view (right gonocoxite I is removed) **E** right gonocoxite I. Abbreviations: *ga.I* – gonapophyse I; *ga.II* – gonapophyse II; *gc.I* – gonocoxite I; *gc.II* – gonocoxite II; *gp* – gonoplacs; *pt.VIII* – ventral paratergite VIII; *s.r* – second ramus; *t.VIII* – tergite VIII; *t.IX* – tergite IX. Scale bars: 1 mm.

***Female external terminalia*** (Fig. 8C–E). Gonocoxites I not fused with paratergites VIII, large, convex, trapezoidal, slightly wider than long, with anterior margins laterally straight, slightly concave medially, medioposterior angles truncated, other angles rounded. Gonocoxites I along their entire medial margins connected to each other by long membrane. Gonapophyses I shaped as small triangular plates located at truncate medioposterior angles of gonapophyses I; they slightly more sclerotised than the latter, with rounded apices. First rami absent. Thin, long, sclerotised, slightly S-shaped band beginning from apex of each gonapophysis I and passing in middle of its ventral membranous wall, and then continuing on ventral wall of gynatrium. Tergite IX oblique downward, roof-shaped, with long dorsal slope and short ventral one; in ventral view, it tapers trapezoidally caudad, with two short tubercles on sides of posterior margin, that being shallowly notched between them. Paratergites IX fused with their median tergite (suture between them retained), small, triangularly tapering anteriorly, articulated with lateral margins of gonocoxites I. Gonocoxites II shaped as rather long, narrow plates tapering towards their anterior and posterior ends; the latter continuing under tergite IX; each gonocoxite I in middle of its lateral margin articulated with posterior limb of gonangulum. Gonapophyses II rather large, membranous, acutely tapering towards narrowly rounded apices directed posteriorly. Second rami distinct, looking like strongly sclerotised and rather wide bands running along lateral margins of gonapophyses II; their posterior ends slightly not reaching apices of the gonapophyses, anterior ends arcuate and connected with anterior ends of gonocoxites II. Each gonoplac short and wide, oval, convex medially and shaped as flat, triangular plate laterally. Both gonoplacs connected to each other by narrow membranous suture into single arcuate structure with convex part facing posteriad. Tergite IX, gonocoxites I, gonapophyses I, and medial parts of gonoplacs covered with setae, some of them semierect. Proctiger membranous, retracted inward under tergite IX, with dorsal and ventral valves reinforced with thin horseshoe-shaped sclerites. Wide, rather short, tapering anteriorly, inner membranous fold, that probably being subrectal gland, located between proctiger and dorsal wall of gynatrium; dorsal wall of this fold connecting posterior ends of gonocoxites II.

**Figure 9.**
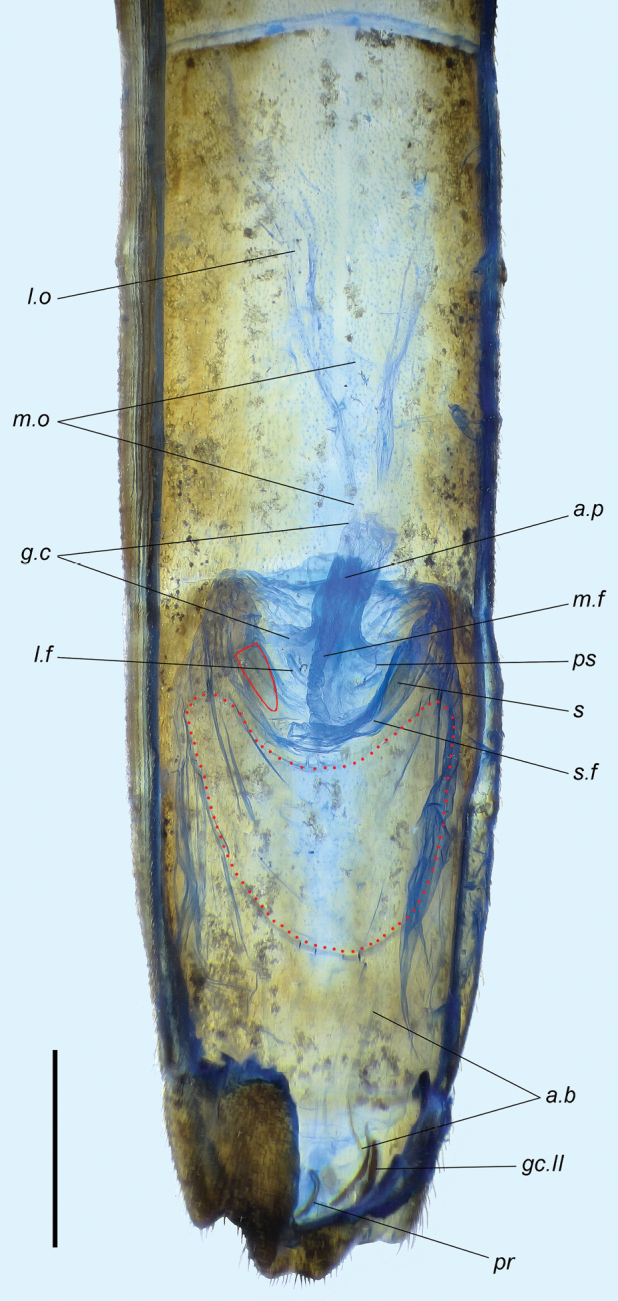
*Rhaphidosomapaganicum* sp. nov., gynatrium in dorsal view, at a wet preparation. Abbreviations: *a.b* – anterior band of gonapophyse I; *a.p* – anterior pouch of gynatrial cone; *g.c* – gynatrial cone; *gc.II* – gonocoxite II; *l.f* – lateral fold of dorsal wall of gynatrium; *l.o* – lateral ectodermal oviduct; *m.f* – median fold of dorsal wall of gynatrium; *m.o* – medial oviduct; *pr* – proctiger; *ps* – supposed duct of pseudospermatheca; *s* – sclerite of semicircular fold (contour of the left sclerite is outlined with a red line); *s.f* – semicircular fold of dorsal wall of gynatrium. Red dotted line outlines the ring sclerite. Scale bar: 1 mm.

***Gynatrium*** (bursa copulatrix) (Fig. 9) shaped as voluminous, longitudinally elongated sac reaching anterior margin of ventrite VII. In extreme base, it narrow, sharply widening anteriorly; lateral walls of its base at the level of anterior ends of gonocoxites II with two large depressions jutting into cavity of gynatrium. Distal to base, gynatrium widening gradually, with subparallel lateral walls in anterior half. Anterior wall almost straight, anteriolateral angles broadly rounded. Ventral wall with very large unpaired ring sclerite shaped like very thin edging, that being oval posteriorly and smoothly concave anteriorly. Dorsal wall of gynatrium in its anterior part forming large, external (protruding into body cavity) semicircular fold; its convex part facing posteriad, anterior ends reaching anterolateral angles of gynatrium. Ventral (anterior) wall of this fold on each side forming rather large longitudinal sclerite with parallel margins, subrectangular anterior end and triangularly narrowed posterior one. Gynatrial cone large, broadly funnel-shaped, flattened dorsoventrally at extreme base, distally cylindrical, directed anteriad, protruding beyond anterior margin of gynatrium; its walls thick in proximal part and thin, with longitudinal folds in extreme distal part. Deep external median fold extending from semicircular fold to approximately middle of gynatrial cone; its walls posteriorly accordion-folded; ante­riorly, this fold forming longitudinal pouch directed anteriad. A pair of small, arcuate folds located posterior to base of gynatrial cone. Just anterior to each of these folds, very thin and rather long thread attaching to dorsal wall of gynatrium [these structures are probably pseudospermathecae, because fragments of a membrane were visible at the distal end of one of the threads, and this membrane is probably part of the destroyed bulb]. Vermiform gland not found. Common oviduct thin-walled, rather long, narrow in place of connection with gynatrial cone, strongly widening anteriorly; ectodermal lateral oviducts rather wide, long, extending beyond anterior margin of ventrite VI.

***Measurements*** (males / female). Body length 19.04–19.89 / 20.23; length of head 3.55–3.78 / 3.58; length of head anterior to transverse sulcus 1.93–2.05 / 2.03; length of head posterior to transverse sulcus 1.73–1.63 / 1.55; width across eyes 0.91–0.95 / 0.93; synthlipsis 0.50–0.53 / 0.50; length of prothorax at midline 1.48–1.70 / 1.58; width of prothorax 1.23–1.30 / 1.28; length of mesothorax 1.28–1.56 / 1.45; width of mesothorax 1.43–1.53 / 1.50; length of metathorax 0.43–0.50 / 0.43; width of metathorax 1.50–1.63 / 1.63; width of abdomen at level of first spiracles 1.13–1.25 / 1.30; length of abdomen 13.40–14.50 / 14.70; length of first antennal segment 6.15–6.50 / 5.50; length of second antennal segment 3.45–3.60 / 3.20; length of third antennal segment 2.55–2.90 / 2.25; length of fourth antennal segment 2.15; total length of labium 3.73–3.93 / 3.75; length of second visible labial segment 2.78–2.98 / 2.78; length of third visible labial segment 0.50–0.55 / 0.53; length of fore coxa 0.78–0.85 / 0.78; length of fore femur 6.70–7.10 / 6.30; length of fore tibia 8.80–9.10 / 8.10; length of middle coxa 0.70–0.78 / 0.73; length of middle femur 5.40–5.90 / 5.70; length of middle tibia 6.70–7.20 / 6.50; length of hind coxa 0.83–0.88 / 0.88; length of hind femur 9.70–10.60 / 9.40; length of hind tibia 11.4–12.6 / 11.3.

####### Distribution and bionomics.

**Figure 10.**
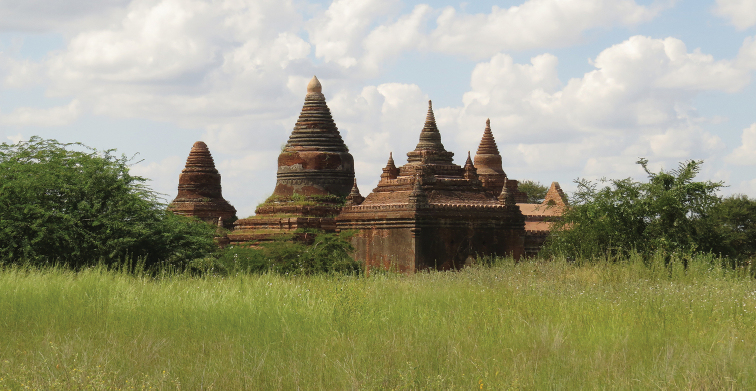
Habitat in the type locality of *Rhaphidosomapaganicum* sp. nov.

The species was found within the Dry zone in central Myanmar, whose climate, according to the classification of Beck et al. (2018), is dry, steppe, hot, with a low average annual rainfall of less than 1,000 mm and a dry season lasting nine months or longer (Gupta 2005). The specimens were collected by sweeping over grass in areas of a herb-grass steppe with individual thorny shrubs and low trees, particularly from the genus *Acacia* Mill., 1754, interspersed with agricultural landscapes and Buddhist religious buildings (Fig. 10).

####### Etymology.

The specific name *paganicum* is a Latin adjective meaning “heathen”; it is given after the type locality that belonged to the Kingdom of Pagan in the XI–XIII centuries AD.

####### Comparison.

Being predominantly Afrotropical, the genus is represented by only four species in the Oriental Region (*Rh.atkinsoni* Bergroth, 1893, *Rh.tuberculatum* Distant, 1904, *Rh.greeni* Distant, 1906, and *Rh.madukaraiensis* Ravichandran et Livingstone, 1994). Four species are also known from Western Asia (*Rh.argillaceum* Horváth, 1929, *Rh.bergevini* Poppius, 1911, *Rh.lutescens* Poppius, 1911, and *Rh.davatchiae* Dispons et Villiers, 1967), and they must be taken into account in comparison with the new species. All these species were described very superficially, more often from one or two specimens of the same sex, and only one of them, *Rh.atkinsoni*, was recently redescribed in sufficient detail (not counting the male and female terminalia) by Pansare et al. (2017). Since I have no material on these species, except for specimens from Iran and Afghanistan, identified by me as *Rh.tuberculatum*, I consider it necessary to compare all the characters given in the original descriptions of these species with those of the new species.

***Differences from Rh.argillaceum*** [described by Horváth (1929: 331) from one male and one immature shrunken female]. In new species, body dark reddish-brown, often with blackish sides, ventral surface of abdomen with a more or less distinct median yellowish stripe, tarsi and last two segments of antennae yellowish brown [*vs.* body argillaceous, head ventrally and thorax laterally except for coxal cavities black]; head 1.18–1.32 times as long as pronotum and mesonotum combined [*vs.* they of equal length]; preocular part of head 1.18–1.30 times as long as postocular part [*vs.* postocular part slightly longer than preocular part]; first antennal segment 0.88–0.93 times in males and 0.87 times in female as long as fore tibia [*vs.* 0.80 times in male]; mesonotum posteriorly with distinct triangular scutellum and vestiges of fore wings [*vs.* mesonotum posteriorly truncate]; abdomen dorsally without distinct tubercles in male, with a pair of long finger-like tubercles on tergite VII and a pair of small rounded tubercles on tergite VI in female [*vs.* abdomen dorsally unarmed in both sexes; but according to Linnavuori (1973), dorsum of abdomen in female with a longitudinal row of three pairs of erect plug-shaped median tubercles]; longer, body length 19.04–19.89 in males, 20.23 in female [*vs.* 18.50 in male, 19.50 in female].

***Differences from Rh.bergevini*** [described by Poppius (1911: 101) from two males]. Body colouration as stated above [*vs.* yellow-grey, thorax (Mittelkörper) sometimes darkened, abdomen (Hinterkörper) black-brown above, sometimes more or less extensively pale, with pale apex, laterally with yellow spots or completely yellow with regularly broken row of black spots, ventral surface brown, with more or less extensive yellow pattern, antennae and legs yellow, extreme apices of tibiae and last article of tarsus black, femora with black ring before apex]; head 1.18–1.32 times as long as pronotum and mesonotum combined [*vs.* head as long as pro- and mesonotum combined]; first antennal segment not thickened towards base, 1.19–1.24 times in males and 1.09 times in female as long as head and pronotum combined [*vs.* slightly thickened towards base, about 0.71 times (etwa 2/7 kürzer) in males as long as head and pronotum combined]; first antennal segment 1.74–1.81 times in males and 1.72 times in female as long as second segment, second and third segments combined about three times as long as fourth segment [*vs.* first segment a little more than three times as long as second one, fourth segment about as long as previous two segments combined]; second segment of rostrum about five times as long as first segment [*vs.* about four times]; pronotum without median carina [*vs.* pronotum in middle with slightly raised longitudinal carina]; tubercles on abdominal tergites as stated above [*vs.* dorsum of abdomen in female with longitudinal row of four pairs of erect plug-shaped median, according to Linnavuori (1973)]. Body length 19.04–19.89 in males [*vs.* 19.50–20.00].

***Differences from Rh.lutescens*** [described by Poppius (1911: 102) from one female]. Body colouration as stated above [*vs.* yellow, some narrow longitudinal lines and small spots on each side of abdominal dorsum, large spot on each side at base of each dorsal segment, sides of head, sides of thorax, transverse band at base of each ventral segment, first antennal segment basally, extreme apex of tibiae and apex of last tarsal segment brown to brown-black; according to Linnavuiri (1973), body testaceous, sides of head and thorax with longitudinal black band, dorsum of abdomen and connexivum with irregular dark pattern, antennae and legs yellowish]; head 1.18–1.32 times as long as pro- and mesonotum combined [*vs.* head about as long as pro- and mesonotum combined]; preocular part of head long, clypeus apically obtuse [*vs.* head short anteriorly, but clearly pointed]; first antennal segment not thickened towards base, 1.19–1.24 times in males and 1.09 times in female as long as head and pronotum combined, last three segments combined 1.33 times as long as first one, second segment 1.21–1.37 times in males and 1.16 times in females as long as third one and together they almost three times as long as fourth segment [*vs.* first segment slightly and gradually thickened towards base, almost 0.71 times as long as head and pronotum combined, last three segments combined hardly shorter than first one, second and third segments about of equal length, together hardly longer than last segment]; mesonotum without sulci [*vs.* mesonotum at base with two longitudinal sulci]; metanotum without middle carina [*vs.* with three longitudinal carinae, one of which slightly curved on each side, and middle one straight]; dorsum of female abdomen along its entire length with three thin carinae and with tubercles as stated above [*vs.* abdominal dorsum in middle with longitudinal carina completely smoothed anteriorly and slightly raised in posterior part, from segment II onwards with tubercle, that being small and simple on segment II, gradually becoming stronger on following segments, with clearly forked, darkened tips, tubercle on genital segment (sic!) divided almost to base]; longer, body length 20.23 in female [*vs.* 20].

***Differences from Rh.davatchiae*** [described by Dispons and Villiers (1967: 1070) from one male]. Body colouration as stated above [*vs.* head brown, with postocular part yellowish dorsally, pronotum brown with yellowish base, meso- and metanotum light brown with yellowish lateral carinae, ventrally thorax brown with yellowish margins of coxal cavities (? le pourtour des hanches), abdomen brown with yellowish base of first visible tergite, base of apical “horn” and small spots on connexivum]; head 2.22–2.51 times in males, 2.27 times in female as long as pronotum [*vs.* head relatively robust, 1.50 times in male as long as pronotum]; preocular part of head 1.18–1.23 times in males, 1.31 times in female as long as postocular part [*vs.* 1.50 times in male]; distance between eye and apex of antenniferous tubercle about 3.50 times as long as eye in dorsal view [*vs.* 2.25 times]; interocular space 2.29–2.47 times in males, 2.35 times in female as wide as eye in dorsal view [*vs.* twice as wide as eye in male]; clypeus without prominence [*vs.* clypeus with short triangular prominence anteriorly]; mesonotum approximately 1.60 times as long as wide, its posterior angles rounded, posterior margin with distinct triangular scutellum and vestiges of fore wings [*vs.* mesonotum subrectangular, almost 1.50 times as long as wide (39:27), its posterior angles obliquely truncated, base concave with outline of median carina; according to the drawing (Dispons and Villiers 1967, fig. 1), posterior margin with medial notch, without distinct scutellum and wing vestiges]; metanotum wider than length, without distinct medial carina, its posterior margin with convex lateral parts, concave medially, with small triangular projection, vestiges of hind wings present [*vs.* metanotum slightly longer than wide, distinctly shorter than mesonotum, with three longitudinal carinae; according to the drawing (Dispons and Villiers 1967, fig. 1), posterior margin uniformly concave, vestiges of wings apparently absent, and what is mistaken for the latter in the description is a continuation of the lateral carina]; tergite VII of male almost three times as long as wide, smoothly and slightly widened anterior to apex, apical carina narrow [*vs.* tergite VII twice as long as wide, with greatest width at level of apical five seventh, where its sides slightly angular; according to the drawing (Dispons and Villiers 1967, fig. 1), apical carina of tergite VII wide]; longer, body length in males 19.04–19.89 [*vs.* 16.00].

***Distinguished from Rh.atkinsoni*** [described based on an unspecified number of specimens (presumably males), redescribed by Pansare et al. (2017) from two males and two females] by following main characters. In male, thorax and abdomen with more or less evenly distributed setae, dorsally without lateral stripes of dense setae; pubescence on abdomen ventrally less dense. Lateral margins of head just posterior to transversal sulcus less convex; entire dorsum of head, including area just posterior to sulcus, almost flat [*vs.* tumescent]. First segment of rostrum noticeably shorter than preantennal part of head [*vs.* almost as long as preantennal region]. Pronotum only slightly convex dorsally [*vs.* tumescent above], its posterior margin slightly convex in middle and concave laterally [*vs.* straight]. Metanotum uniformly convex in posterior part of disc, without carina [*vs.* with median raised area or blunt carina along its length], with posterior margin convex laterally, concave in middle [*vs.* sinuate]. Ventral parts of posterior lobe of prothorax (epimera) contiguous [*vs.* not meeting each other, with a noticeable gap in the midline]. Thoracic segments dorsally and ventrally without granules [*vs.* provided with few dark brown granules]. Boundary between metasternite and abdominal ventrite II distinct [*vs.* indistinct]. In males, medial part of abdominal tergites not shining, all tergites without mid-dorsal tubercles [*vs.* median part of each tergite shining; third and fourth tergites with small mid-dorsal tubercle]; posterior margin of tergite VI in middle slightly elevated, that of tergite V elevated even less strongly, these areas not shining [*vs.* posterior border of tergites III–VI medially slightly elevated as small shining tubercle]. In female, tergite V in middle of posterior margin without distinct tubercle, tergite VI with two small rounded tubercles, tergite VII with two large finger-like tubercles located on strongly raised base [*vs.* tergite V with small mediodorsal tubercle at posterior border, tergite VI with a pair of small tubercles, and tergite VII with a pair of small, posteriorly directed blunt tubercular projections on either side of midline]. Posterior margins of laterotergites and median tergite VIII with rather long triangular projections [*vs.* posterior margins of laterotergites rounded, posterior margin of tergite with very short, smoothed projections]. Male and female terminalia are not well described for comparison. Pygophore more smoothly convex ventrally in lateral view, with more parallel lateral margins and two denticles of medial process at apex in ventral view [*vs.* sharply and almost triangularly convex ventrally, rhomboid-like widening before apex, the latter with one triangular denticle in ventral view, according to the photographs (Pansare et al. 2017, figs 32–34)]. Paramere less strongly curved at base and more strongly at slightly widening apex [*vs.* apex uniformly narrow, straight, according to the photographs (Pansare et al. 2017, figs 32–34)]. Shorter, body length 19.04–19.89 in males, 20.23 in female [*vs.* 25.10 in males, 23.50 in females].

***Differences from Rh.tuberculatum*** [described by Distant (1904: 330) based on an unspecified number of specimens, presumably from female(s)]. Body colouration as stated above [*vs.* pale greyish, tibiae and last two joints of antennae pale ochraceous]; thorax dorsally without small tubercles [*vs.* with marginal series of small tubercles on each side of thorax above, a number of small discal tubercles to pronotum]; only tergites V and VI in females with paired tubercles [*vs.* two prominent tubercles at posterior margins of third (IV), fourth (V), fifth (VI), and sixth (VII) abdominal segments]; apex of head tapered, obtuse [*vs.* distinctly porrectly spinous]; first antennal segment 1.21–1.46 times in males, 1.20 times in female as long as distance from eye to base of thorax [*vs.* first joint of antenna about as long as from eyes to base of thorax]; head very slightly swollen laterally behind eyes [*vs.* head distinctly tumid behind eyes]; pronotum slightly convex, its anterior angles angularly rounded [*vs.* convexly tumid, its anterior angles sinuously produced]; vestiges of both pairs of wings present [*vs.* absent in available for me specimens]. Shorter, body length 19.04–19.89 in males, 20.23 in female [*vs.* 23].

***Differences from Rh.greeni*** [described by Distant (1906: 367) based on an unspecified number of specimens, presumably from male(s)]. Body colouration as stated above [*vs.* piceous black, intermediate and hind tibiae dull ochraceous, tarsal claws piceous, antennae pale castaneous brown, abdomen above pale piceous brown, a central longitudinal fascia and the lateral margins black]; preocular part of head 1.18–1.30 times as long as postocular part [*vs.* head elongate, ante- and postocular portions almost subequal in length]; first antennal segment 1.10–1.12 times in males and 0.96 times in female as long as middle femora [*vs.* antennae first joint as long as middle femora]; second antennal segment 1.21–1.37 times in males, 1.42 times in female as long as third one and they combined approximately equal to first segment [*vs.* second and third joints subequal in length and each considerably shorter than first]; fore femora longer than middle femora [*vs.* fore and middle femora subequal in length]; hind femora 0.71–0.78 times in males, 0.64 times in female as long as abdomen, hind tibia 0.87–0.91 times in males, 0.77 times in female as long as abdomen [*vs.* hind femora a little shorter and hind tibiae a little longer than abdomen]. Shorter, body length 19.04–19.89 in males, 20.23 in female [*vs.* 25].

***Differences from Rh.madukaraiensis*** [described by Ravichandran and Livingstone (1994: 106) from males (and presumably female)]. Body colouration as stated above [*vs.* concolourous, griseous, antennae concolourous, castaneous, connexivum (abdomen?) ventrally griseous, with a median longitudinal line]; preocular part of head 1.18–1.30 times as long as postocular part [*vs.* anteocular and postocular areas subequal in length]; postocular part of head slightly tapering to base [*vs.* tumid throughout]; second antennal segment 1.21–1.38 times in males, 1.42 times in female as long as third segment and 1.6 times as long as fourth one, third and fourth segments combined 0.70 times as long as second segment [*vs.* pedicel and flagellar segments equal]; pronotum slightly convex dorsally, its lateral margins almost parallel posteriorly and converging anteriorly, pubescence as on other segments of thorax [*vs.* pronotum slightly globose, spotted, almost bare]; mesonotum trapezoidal, rather weakly convex [*vs.* nodule like]; matanotum without medial carina, its posterior margin with convex lateral parts, distinctly concave medially, with small medial prominence [*vs.* metanotum medially carinate, posteriorly obscurely concave]; abdominal tergites without distinct tubercles in male, with small paired rounded tubercles on tergite VI and long tubercles on tergite VII in female [*vs.* second (III), third (IV), fourth (V) and fifth (VI) segments dorsally with a forked tubercle]. Shorter, body length 19.04–19.89 in males [*vs.* 20 in males].

####### Note.

According to the description of *Rh.madukaraiensis*, it differs from *Rh.tuberculatum* “by the total absence of thoracic tubercles, cephalic spine and by the obscure development of scutellum, wing pads and mesonotal median carina”. In the specimens of *Rh.tuberculatum* available to the first author, vestiges of the fore and hind wings are completely absent. The relief triangular structures in the posterior angles of the metanotum, which can be mistaken for vestiges of the hind wings, are in fact lateral carinae. Scutellum of *Rh.tuberculatum* is very small, much smaller than that of the new species; the mesonotal median carina is absent in both of these species. According to the description of *Rh.madukaraiensis*, the type series (holotype and several paratypes) consists of males only, but the listed characters of the sculpture of abdominal tergites and the phrase “behind the fifth segment the abdomen abruptly terminates” correspond to the female. The median tergite and paratergites VIII in the female of the new species have rather large triangular prominences on the posterior margins.

## ﻿Chromosomal analysis

### Karyotypes

#### *Rhaphidosomapaganicum* sp. nov.

**Figure 11.**
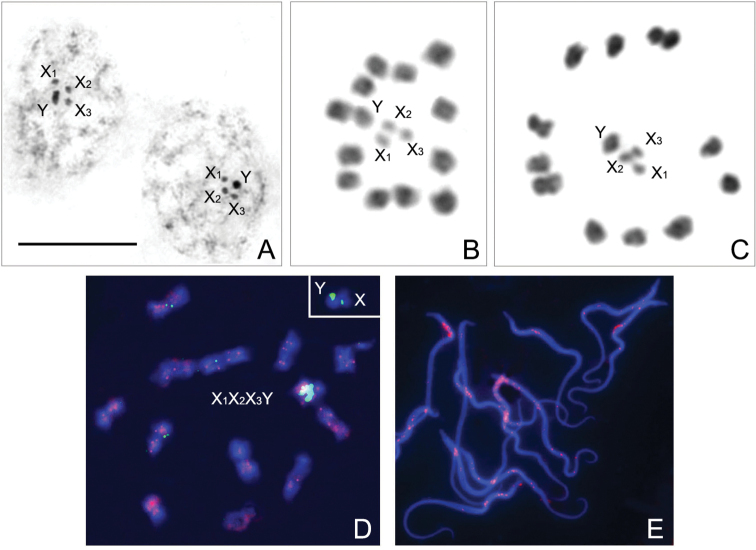
Male meiotic chromosomes of *Rhaphidosomapaganicum* sp. nov., n=12AA+X_1_X_2_X_3_Y (2n=24A+ X_1_X_2_X_3_Y) **A** first prophase, early condensation stage (chromosomes X_1_X_2_X_3_ are located in a train and the Y is located slightly away from them) **B, C** metaphase I (sex chromosomes X_1_X_2_X_3_Y are lying side by side inside the ring formed by autosomal bivalents) **D** hybridisation signals of *18S rDNA*-FISH (green) and (TTAGG)*_n_*-FISH (red) are visible on Y and one of the X-chromosomes (small green signals are also seen in some autosomal bivalents, but they proved to be not specific), and on both ends of each chromosome, respectively; **Inset**, Y and an X-chromosome with *18S rDNA* signals **E** (TTAGG)*_n_*-FISH-signals are visible in spermatids. Scale bar: 10 μm.

Based on the analysis of the only male (paratype) available for such a study, we conclude that the male chromosome complement of the species comprises 12 pairs of autosomes and a multiple sex chromosome system, X_1_X_2_X_3_Y, i.e., n=16(12AA+X_1_X_2_X_3_Y) and 2n=28(24A+X_1_X_2_X_3_Y). The autosomal bivalents make up a decreasing size range; of the four sex chromosomes, Y is the largest, and all three X-chromosomes are approximately the same size (Fig. 11A–D). At early condensation stage of the first meiotic prophase, sex chromosomes are grouped together as four heteropycnotic bodies, with all X-chromosomes located in a train and the Y placed away from them (Fig. 11A). At metaphase I (MI), the sex chromosomes appear clearly separated from one another; they are lying side by side inside the ring formed by autosomal bivalents and show no visible physical connection with each other (Fig. 11B, C). FISH with two repetitive DNA probes, *18S rDNA* and telomeric (TTAGG)_*n*_, visualised, on the one hand, clusters of *rRNA* genes on the Y-chromosome and on one of the X-chromosomes, and, on the other hand, the (TTAGG)_*n*_-signals at both ends of each chromosome (Fig. 11D) and also in spermatids (Fig. 11E).

As mentioned in Introduction, the most common karyotype of Harpactorinae includes 2n=28(24A+X_1_X_2_X_3_Y), and *Rh.paganicum* sp. nov., thus, shares this modality with regard to both the number of autosomes and the sex chromosome system. Previously, there was only one species in the genus *Rhaphidosoma* with a known (standard) karyotype, *Rh.truncatum* Jeannel, 1914. According to Kpordugbe (1979), this species possesses the karyotype of 2n=26(24A+XY), i.e., *Rh.truncatum* has 24A as *Rh.paganicum* but, unlike it, a simple sex chromosome system. We can assume that the multiple sex chromosome system of *Rh.paganicum* arose because of the fragmentation of the X-chromosome in the ancestral karyotype with the XY-system. This character is interesting in light of the fact that the new species seems to have several more primitive morphological characters than in *Rh.truncatum*, in particular, distinct vestiges of the fore and hind wings, which are not mentioned for the latter in the description by Jeannel and are not shown in his drawing (Jeannel 1919, fig. 60).

#### *Polididusarmatissimus* Stål, 1859

**Material examined. Thailand**, **Mae Hong Son Prov.**, Pai Distr., near Pai Town, 19°21'25.8"N, 98°27'01.0"E, 12,13.XI.2019, at light, D.A. Gapon leg., 8 males (ZISP).

**Figure 12.**
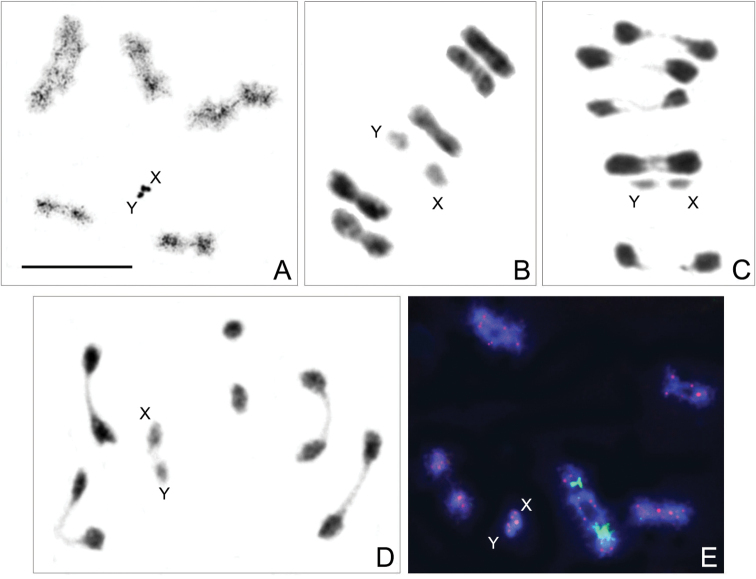
Male meiotic chromosomes of *Polididusarmatissimus* Stål, 1859, n=5AA+XY (2n=10A+XY) **A** diplotene/diakinesis; heteropycnotic X- and Y-chromosomes form a pseudo-bivalent **B, C, D** metaphase I/anaphase I transition; X- and Y-chromosomes form a pseudo-bivalent **E** hybridisation signals of *18S rDNA*-FISH (green) and (TTAGG)*_n_*-FISH (red) are visible on AAI and on both ends of each chromosome, respectively. Scale bar: 10 μm.

Based on the analysis of eight males, we conclude that the male chromosome complement of *P.armatissimus* comprises five pairs of autosomes and a simple sex chromosome system, XY, i.e., n=7(5AA+XY) and 2n=12(10A+XY) as previously noted by Banerjee (1958). The autosomal bivalents are clearly larger than in *Rh.paganicum* sp. nov. and make up a decreasing size series. The X- and Y-chromosomes are close to each other in size being at the same time significantly smaller than autosomes (Fig. 12A–E). At the first meiotic prophase, sex chromosomes are grouped together and appear as two heteropycnotic bodies (Fig. 12A). The autosomes are associated by one or, in some bivalents, two chiasmata, and the sex chromosomes form a pseudo-bivalent (Fig. 12B–D). FISH with probes for *18S rDNA* and (TTAGG)_*n*_ visualised *rDNA* clusters located on the largest autosomal bivalent (AA1) and positive (TTAGG)*_n_*-signals at both ends of each chromosome (Fig. 12E).

It should be noted that *P.armatissimus* has the lowest number of autosomes (10A) ever recorded in Harpactorinae. It was previously shown that males of this species originated from Japanese and Indian (Calcutta) populations also have 2n=12(10A+XY), although males from northwest India were reported to have 2n=14(12A+XY) (see for references Ueshima 1979).

### Telomeres

Telomeres of both *Rh.paganicum* sp. nov. and *P.armatissimus* have clearly demonstrated presence of the TTAGG repeat, which constitutes the first report of the (TTAGG)_*n*_ telomere motif in the tribe Raphidosomatini and in the genus *Polididus* (Harpactorini). This so-called “insect” repeat is considered an ancestral DNA motif of insect telomeres (Frydrychová et al. 2004; Kuznetsova et al. 2020). Recent research has revealed several examples of heterogeneity for presence/absence of the (TTAGG)_*n*_ motif in different insect groups (see for references Kuznetsova et al. 2020), and such heterogeneity was also found in Heteroptera, including the infraorder Cimicomorpha. Thus, no hybridisation signals were detected in the families Nabidae, Miridae, Tingidae, and Cimicidae in the experiments using FISH with the (TTAGG)*_n_* telomeric probe; moreover, the TTAGG telomeric sequence was not found in the sister to Cimicomorpha infraorder Pentatomomorpha (see for references Grozeva et al. 2019). On the contrary, the telomere motif (TTAGG)*_n_* appears to be characteristic of all studied species of the family Reduviidae (in total, eight species and six genera of the largest reduviid subfamilies, Triatominae and Harpactorinae). These species are as follow: *Dipetalogastermaxima* Uhler, 1894, *Rhodniusprolixus* Stål, 1859, *Triatomainfestans* Klug, 1834, and *T.dimidiata* Latreille, 1811 from Triatominae (Pita 2016), as well as *Rhaphidosomapaganicum*, *P.armatissimus*, *Rhynocorispunctiventris* (Herrich-Schäffer, 1846), and *Rh.iracundus* (Poda, 1761) from Harpactorinae (Grozeva et al. 2019; present study). Although the available data are still sparse, they allow suggestion that the (TTAGG)*_n_* motif is characteristic of the whole family Reduviidae. Moreover, it was hypothesised that an ancestor of the branch Cimicomorpha + Pentatomomorpha possessed this motif that retained in the Reduviidae but was then repeatedly lost by other families of this branch (Grozeva et al. 2019). This character together with other plesiomorphic characters such as the presence of the first pair of abdominal spiracles, the spermatophore insemination, and a place of fertilisation in the ectodermal parts of the female oviducts confirms the hypothesis (Schuh and Štys 1991; Schuh et al. 2009) of the basal position of the superfamily Reduvioidea on the phylogenetic tree of Cimicomorpha.

### 18S rDNA

We found that the genes encoding the major *rRNA*s were located differently in *Rh.paganicum* sp. nov. and *P.armatissimus*. These species have different numbers of autosomes (24A and 10A, respectively) and different sex chromosome systems (X_1_X_2_X_3_Y and XY, respectively); however, there seems to be no correlation between these two characters and the *rDNA* chromosomal location in Harpactorinae (Table 1). Table 1 summarises the location of the *18S rDNA* clusters in nine species of Harpactorinae studied until now. These species belong to six genera and three of the seven recognised tribes, Apiomerini (1 species), Harpactorini (7 species), and Rhaphidosomatini (1 species). The species analysed present three (10A, 12A and 24A) of the six numbers of autosomes (excluding 18A, 20A, and 22A) and two (XY and X_1_X_2_X_3_Y) of the five sex chromosome systems (excluding X_1_X_2_Y, X_1_X_2_X_3_X_4_Y and X_1_X_2_X_3_X_4_X_5_Y) described in Harpactorinae. The studied species are still very few. Despite this, we can identify four distinct patterns of *rDNA* location in Harpactorinae. First, *rDNA* clusters can be located on the largest pair of autosomes (found in three species with XY and different autosome numbers). Second, they can be located on both sex chromosomes (in one species with XY). Third, they can be located on X-chromosome (in one species with XY). Fourth, they can be located on Y-chromosome and one of the X-chromosomes (in all four species with 24A and X_1_X_2_X_3_Y). We can assume that the subfamily Harpactorinae has a very high level of *rDNA* location diversity that is comparable to the diversity observed in the much better studied subfamily Triatominae (see Introduction). Moreover, it is very likely that such diversity is characteristic of the family Reduviidae as a whole.

**Table 1.** Chromosomal location of genes for *18S rRNA* in karyotypes of nine species of the subfamily Harpactorinae studied to date.

**N**	**Species**	**2n**	**Location of *18S rDNA***	**Reference**
	**Tribe Apiomerini**			
1	*Apiomeruslanipes* (Fabricius, 1803)	24(22A+XY)	X and Y	Bardella et al. 2014
	**Tribe Harpactorini**			
2	*Cosmoclopiusnigroannulatus* (Stål, 1860)	28(24A+X_1_X_2_X_3_Y)	Y and one of Xs	Bardella et al. 2014
3	*Montinaconfusa* (Stål, 1859)	14(12A+XY)	AAI	Bardella et al. 2014
4	*Polididusarmatissimus* Stål, 1859	12(10A+XY)	AAI	Present paper
5	*Repiptaflavicans* (Amyot et Serville, 1843)	20(18A+XY)	One of sex chromosomes	Bardella et al. 2014
6	*Rhynocorisiracundus* (Poda, 1761)	28(24A+X_1_X_2_X_3_Y)	Y and one of Xs	Grozeva et al. 2019
7	*Rhynocorispunctiventris* (Herrich-Schäffer, 1846)	28(24A+X_1_X_2_X_3_Y)	Y and one of Xs	Grozeva et al. 2019
8	*Zeluslaticornis* (Herrich-Schäffer, 1853)	26(24A+XY)	AAI	Bardella et al. 2014
	**Tribe Rhaphidosomatini**			
9	*Rhaphidosomapaganicum* sp. nov.	28(24A+X_1_X_2_X_3_Y)	Y and one of Xs	Present paper

### Testes

We found that the testes of *Rh.paganicum* sp. nov. and *P.armatissimus* males are located laterally at the sides of the alimentary tract, approximately in the middle of the abdominal cavity. The species have seven and nine elongated follicles per testis, respectively. In *Rh.paganicum*, the follicles have similar length and width; they are arranged side by side being connected by bases. In *P.armatissimus*, the structure of the testis turned out to be more complex. The follicles are folded three times onto each other and wrapped with a reddish peritoneal sheath. Moreover, they form two groups, one consisting of seven long and wide follicles and the other consisting of two smaller follicles. The same testis structure, although with a different ratio of large and small follicles, is also characteristic of some other species of Reduviidae (Louis and Kumar 1973; Freitas et al. 2007). In Rhaphidosomatini, all the three species studied so far (*Rhaphidosomaoccidentale*, *Lopodytesarmatus*, and *L.quadrispinosus*) also have seven follicles per testis (Louis and Kumar 1973) and it is so with other Harpactorinae species (e.g. Gomes et al. 2013). Moreover, the testis comprising seven follicles seems to be the plesiomorphic condition for Reduviidae, because this number was found in many species throughout the group (Louis and Kumar 1973 and references therein), and for Heteroptera as a whole (Akingbohungbe 1983). Since this number corresponds to the number of pre-genital segments in adult males, it is considered as the initial character state in insects in general (Sharov 1966). The initial number can decrease or increase in oligomerisation or polymerisation processes, which proceed independently in different Heteroptera lineages (Pendergrast 1957).

## ﻿Author contributions

Conception and design of the project, D.G. and V.K.; material collection and description of the new species, D.G.; cytogenetic study, V.K. and A.M-N.; writing the paper, D.G. and V.K.; final approval of the version to be published, all authors.

## Supplementary Material

XML Treatment for Rhaphidosoma paganicum
